# Supplementary material for: Creating amphiphilic porosity in two-dimensional covalent organic frameworks via steric-hindrance-mediated precision hydrophilic-hydrophobic microphase separation
Source: Nat Commun. 2024 Jan 24;15:698. doi: 10.1038/s41467-024-44890-3 (PMC10808405; doi:10.1038/s41467-024-44890-3)
Supplement: Supplementary file 1 — Supplementary Information [file 41467_2024_44890_MOESM1_ESM.pdf]

Supplementary Information for

Creating Amphiphilic Porosity in Two-Dimensional Covalent Organic Frameworks via Steric-Hindrance-Mediated Precision Hydrophilic-Hydrophobic Microphase Separation

Jiang *et al.*

Shu-Yan Jiang,[1,2] Zhi-Bei Zhou,[1,2] Shi-Xian Gan,[1] Ya Lu,[1] Chao Liu,[1] Qiao-Yan Qi,[1] Jin Yao,[1]* and Xin Zhao[1,2]*

[1]Key Laboratory of Synthetic and Self-Assembly Chemistry for Organic Functional Molecules, Center for Excellence in Molecular Synthesis, Shanghai Institute of Organic Chemistry, Chinese Academy of Sciences, 345 Lingling Road, Shanghai 200032, China.

[2] University of Chinese Academy of Sciences, Beijing 100049, China.

Table of Contents

Section A. Instruments and methods. S1

Section B. Synthesis of the monomers and COFs. S3

Section C. Characterization of COF-Bu, COF-Ph and COF-Na. S10

Section D. Characterization of the COF-Bu and COF-Ph membranes. S19

Section E. Characterization of COF-TAB-Hex and SIOC-COF. S21

Section F. Characterization of the COF-TAB-Hex, SIOC-COF, and POP-Bu membranes. S26

Section G. SEM and contact angle images of the membranes. S28

Section H. Fractional atomic coordinates of the COFs. S31

Section I. ^{1}H NMR and ^{13}C NMR spectra...S41

Supplementary References. S46

Section A. Instruments and methods.

Fourier transform infrared spectroscopy (FT-IR)

Fourier transform infrared spectroscopy (FT-IR) was carried out with a Perkin-Elmer PE-983 spectrometer. The samples were fully dried prior to data collection.

Nuclear magnetic resonance (NMR) spectroscopy

For solution phase NMR, ^{1}H NMR and ^{13}C NMR were collected by a JEOL 400 M or an Agilent 500 M instrument.

Solid-state ^{13}C cross-polarization/magic angle spinning (CP/MAS) spectra were collected on Agilent DD2 600 Solid system. ^{1}H-^{13}C CP/MAS experiment using a 3.2 mm HFXY MAS probe and a 3.2 mm ZrO_2 rotor, the cross-polarization time was 1 ms, the cycle delay was set to 2 s, and the ^{13}C chemical shift was calibrated using adamantane (38.56 ppm).

Scanning electron microscopy (SEM)

Scanning electron microscopy was carried out using a XL30 FEG and ZEISS GeminiSEM 300 scanning electron microscope. The samples were dispersed over a slice of conductive adhesive adhered to a flat copper platform sample holder and then coated with gold using a sputter 9 coater (ambient temperature, 85 torr pressure in a nitrogen atmosphere, sputtered for 30 s from a solid gold target at a current of 30 mA) before being submitted to SEM characterization.

Transmission electron microscopy (TEM)

Transmission electron microscopy was performed on a JEOL JEM-2100 instrument. Before the test, the sample powder was dispersed in ethanol to form a suspension. The samples were dispersed over the carbon coated cooper girds with ethanol as solvent.

Thermal gravimetric analysis (TGA)

Thermal gravimetric analyses were carried out on Waters TGA Q500 by heating the samples from 20 to 900 ^oC under nitrogen atmosphere at a heating rate of 10 ^oC /min.

Powder X-ray diffraction (PXRD)

Powder X-ray diffraction measurements were carried out with a PANalytical X' Pert Powder system using monochromated Cu/Kα (λ = 0.1542 nm). The sample was spread on the square recess of XRD sample holder as a thin layer.

Nitrogen adsorption-desorption isotherm measurements

The measurements were carried out using a *Quantachrome autosorb iQ* automatic volumetric instrument. Before gas adsorption measurements, the as-prepared samples (~30 mg) were washed with tetrahydrofuran (THF) for 4 h. The samples were activated by degassing at 120 ^oC for 5 h and used for gas adsorption measurements from 0 to 1 atm at 77 K. The Brunauer-Emmett-Teller (BET) method was utilized to calculate the specific surface areas. By using the non-local density functional theory, the pore size distributions were derived from the sorption data.

Structural modeling and powder X-ray diffraction analysis

Structural modeling was carried out using the Materials Studio 7.0. The predicted structures with eclipsed (AA) and staggered (AB) stacking models were firstly optimized in geometry optimizations by the Forcite molecular dynamics module method, after which the simulated PXRD patterns were determined by the Reflex module. The Pawley refinement of the experimental PXRD was conducted by the Reflex module.

Section B. Synthesis of the monomers and COFs.

Synthesis of 2-butoxy-5-hydroxyterephthalaldehyde (TPA-Bu)

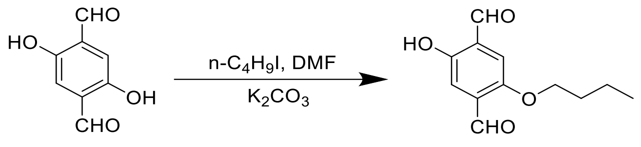

A mixture of DHTA (1.0 g, 6.0 mmol) and K_2CO_3 (500 mg, 3.6 mmol, 0.6 eq) in DMF (50 mL) was degassed through three freeze–pump–thaw cycles under an argon atmosphere and was stirred at room temperature for 0.5 h. 1-iodobutane was then dropped into the mixture and was stirred at 50 °C for about 4 h. After being cooled to room temperature, saturated ammonium chloride solution was added to quench the reaction and the mixture was extracted with dichloromethane. The organic phase was washed consecutively with water and saturated ammonium chloride solution, dried over anhydrous $MgSO_4$, filtered and evaporated. The crude product was purified by flash column chromatograph (PE: EA = 10: l) to give TPA-Bu as yellow powder (481 mg, 35.9 %).

^{1}H NMR (400 MHz, DMSO-d_6) δ 10.55 (s, 1H), 10.36 (s, 1H), 10.34 (s, 1H), 7.34 (s, 1H), 7.29 (s, 1H), 4.09 (t, J = 6.4 Hz, 2H), 1.76 - 1.72 (m, 2H), 1.49 - 1.43 (m, 2H), 0.94 (t, J = 7.6 Hz, 3H). ^{13}C NMR (101 MHz, DMSO-d_6) δ 190.0, 189.2, 154.1, 153.4, 129.5 127.0, 115.5, 111.7, 68.6, 30.6, 18.7, 13.7. FT-IR (cm^{-1}): 3268.3, 2957.7, 2871.2, 1674.6, 1621.9, 1570.3, 1485.7, 1469.7, 1454.4, 1394.8, 1350.9, 1289.4, 1222.1, 1199.2, 1145.2, 1117.3, 1038.0, 1000.1, 912.7, 885.3, 871.2, 801.3, 753.4, 699.4. LRMS (EI): m/z 222 $[M]^+$. HRMS (EI Positive Ion Mode): Calcd. for $C_{12}H_{14}O_4$ $[M]^+$: 222.0887. Found: 222.0888.

Synthesis of 2- hexyloxy-5-hydroxyterephthalaldehyde (TPA-Hex)

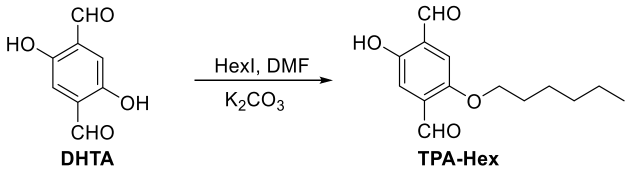

A mixture of DHTA (1.0 g, 6.0 mmol) and K_2CO_3 (1.0 g, 7.2 mmol, 1.25 eq) in DMF (50 mL) was degassed through three freeze–pump–thaw cycles under an argon atmosphere and then was stirred at room temperature for 0.5 h. 1-iodohexane was then dropped into the mixture and was stirred at 50 °C for about 4 h. After being cooled to room temperature, HCl (aq. 2 M) was added to quench the reaction and the mixture was extracted with ethyl acetate. The organic phase was washed consecutively, dried over anhydrous $MgSO_4$, filtered and evaporated. The crude product was purified by flash column chromatograph (PE: EA = 5: l) to give TPA-Hex as yellow powder (530 mg, 35.2 %).

^{1}H NMR (400 MHz, DMSO-d_6) δ 10.55 (s, 1H), 10.36 (s, 1H), 10.34 (s, 1H), 7.34 (s, 1H), 7.29 (s, 1H), 4.08 (t, J = 6.4 Hz, 2H), 1.79 - 1.72 (m, 2H), 1.46 - 1.40 (m, 2H), 1.33 - 1.29 (m, 4H), 0.87 (t, J = 7.2 Hz, 3H). ^{13}C NMR (101 MHz, DMSO-d_6) δ 190.0, 189.2, 154.1, 153.4, 129.5, 127.0, 115.5, 111.7, 68.9, 30.9, 28.4, 25.1, 22.0, 13.9.

FT-IR (cm^{-1}): 3282.0, 2942.4, 2868.2, 1677.4, 1635.0, 1573.6, 1484.4, 1470.4, 1450.9, 1395.8, 1341.1, 1308.4, 1224.1, 1145.4, 1122.5, 1058.5, 1019.0, 995.4, 878.4, 804.5, 755.4, 722.9. LRMS (EI): *m/z* 250 $[M]^+$. HRMS (EI Positive Ion Mode): Calcd. for $C_{14}H_{18}O_4$ $[M]^+$: 250.1200. Found: 250.1197.

Synthesis of Benzyl iodide

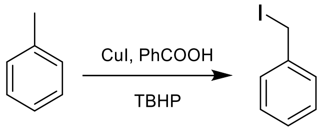

To a stirred mixture of benzoic acid (244.2 mg, 2.0 mmol) and CuI (762.0 mg, 4.0 mmol) in toluene (10 mL) was added tert-butyl hydroperoxide (1.1 mL, 5.5 M in decane) and continuous stir was kept at 80 °C for 5 h. The reaction was quenched by adding CH_2Cl_2 (20 mL) and $Na_2S_2O_3$ (0.6 g), then dried over anhydrous Na_2SO_4. After filtration, the filtrate was concentrated under reduced pressure. The residue was purified by column chromatography and eluted with petroleum ether to afford benzyl iodide as pale yellow liquid (390 mg, 89.7%).

^{1}H NMR (400 MHz, $CDCl_3$-*d*) δ 7.39-7.37 (m, 2H), 7.31-7.24 (m, 3H), 4.46 (s, 2H). The spectrum is consistent with the data previously reported literature.[1]

Synthesis of 2-(Iodomethyl)naphthalene

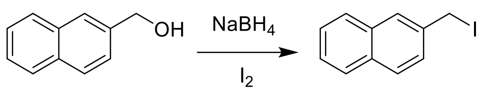

Under N_2 atmosphere, 1,4-dioxane (25 mL) was added into a mixture of naphthalen-2-ylmethanol (2.0 g, 12.6 mmol) and $NaBH_4$ (0.48 g, 12.6 mmol), followed by stirring at 80 °C for 30 minutes. After cooling, iodine (3.2 g, 12.6 mmol) was added into the mixture under N_2. After stirring at 60 °C for 12 hours, distilled water (60 mL) was added to quench the reaction. The crude product was extracted with ethyl acetate and the combined organic phase was dried over anhydrous Na_2SO_4, followed by filtration and concentration by rotary evaporator. The residue was purified by flash column chromatograph (petroleum ether) to give a white solid (1.56 g, 46%).

^{1}H NMR (400 MHz, $CDCl_3$-*d*) δ 7.83-7.78 (m, 4H), 7.49-7.46 (m, 3H), 4.64 (s, 2H). The spectrum is consistent with the data previously reported in literature.[1]

Synthesis of 2-phenoxy-5-hydroxyterephthalaldehyde (TPA-Ph)

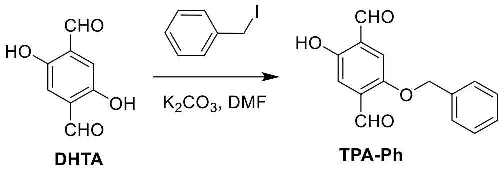

A mixture of DHTA (300 mg, 1.8 mmol) and K_2CO_3 (248.4 mg, 1.8 mmol) in DMF (50 mL) was degassed through three freeze–pump–thaw cycles under an argon atmosphere and then was stirred at 0 °C for 0.5 h. Benzyl iodide (392.4 mg, 1.8 mmol) diluted in anhydrous DMF (10 mL) was then slowly dropped into the mixture and the mixture was stirred at 0 °C for about 4 h. Then, 12 mL HCl (aq. 1 M) was added to quench the reaction and the solvent was removed by rotary evaporation. The crude product was purified by flash column chromatograph (PE: EA = 20: l) to give TPA-Ph as yellow powder (118.7 mg, 25.8 %).

^{1}H NMR (400 MHz, DMSO-d_6) δ 10.63 (s, 1H), 10.40 (s, 1H), 10.35 (s, 1H), 7.51-7.49 (d, J = 7.2 Hz, 2H), 7.46 (s, 1H), 7.42-7.39 (t, J = 7.4 Hz, 2H), 7.36-7.34 (d, J = 6.8 Hz, 1H), 7.32 (s, 1H), 5.26 (s, 2H). ^{13}C NMR (101 MHz, DMSO-d_6) δ 189.2, 188.6, 153.8, 152.4, 135.9, 129.3, 127.9, 127.4, 126.9, 126.3, 115.1, 111.8, 69.9. LRMS (EI): *m/z* 256 $[M]^+$. HRMS (EI Positive Ion Mode): Calcd. for $C_{15}H_{12}O_4$ $[M]^+$: 256.0730. Found: 256.0735.

Synthesis of 2-(naphthalen-2-yloxy) -5-hydroxyterephthalaldehyde (TPA-Na)

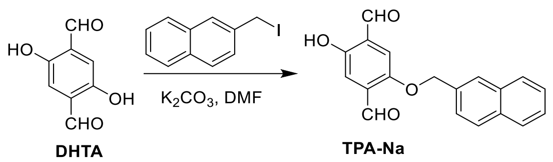

A mixture of DHTA (300 mg, 1.8 mmol) and K_2CO_3 (248.4 mg, 1.8 mmol) in DMF (50 mL) was degassed through three freeze–pump–thaw cycles under an argon atmosphere and then was stirred at 0 °C for 0.5 h. 2-(Iodomethyl)naphthalene (530.6 mg, 1.98 mmol) diluted in anhydrous DMF (10 mL) was then slowly dropped into the mixture and the mixture was stirred at 0 °C for about 4 h. Then, 12 mL HCl (aq. 1 M) was added to quench the reaction and the solvent was removed by rotary evaporation. The crude product was purified by flash column chromatograph (PE: EA = 20: l) to give TPA-Ph as yellow powder (50 mg, 9.1 %).

^{1}H NMR (400 MHz, DMSO-d_6) δ 10.64 (s, 1H), 10.46 (s, 1H), 10.35 (s, 1H), 8.04 (s, 1H), 7.96-7.92 (m, 3H), 7.65-7.63 (d, J = 9.2 Hz, 1H), 7.54-7.51 (m, 3H), 7.34 (s, 1H), 5.43 (s, 2H). ^{13}C NMR (101 MHz, DMSO-d_6) δ 189.2, 188.6, 153.9, 152.4, 133.5, 132.2, 132.0, 129.3, 127.6, 127.3, 127.0, 126.3, 125.7, 125.6, 125.6, 124.9, 115.1, 111.9, 70.0. LRMS (EI): *m/z* 306 $[M]^+$. HRMS (EI Positive Ion Mode): Calcd. for $C_{19}H_{14}O_4$ $[M]^+$: 306.0887. Found: 306.0890.

Procedure for the preparation of COF-Bu

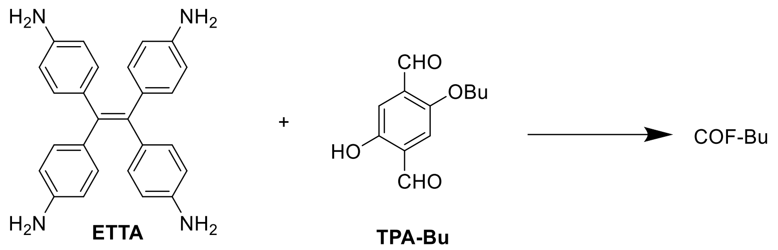

A mixture of 4,4',4'',4'''-(ethene-1,1,2,2-tetrayl)tetraaniline (ETTA, 70.0 mg, 0.178 mmol), 2-butoxy-5-hydroxyterephthalaldehyde (TPA-Bu, 79.3 mg, 0.357 mmol), and 1,4-dioxane (3 mL) in a glass ampoule was sonicated for 10 min and then acetic acid (aq., 6 M, 0.3 mL) was added. The ampoule was sealed after being degassed in a liquid nitrogen bath for 5 min, warmed to room temperature and then kept at 120 ^{o}C without disturbance for 3 days to yield an orange solid. After being cooled to room temperature, the solvent was decanted and the solid was washed with dichloromethane and acetone for 3 times and then dried under dynamic vacuum at 120 ^{o}C for 2 h to afford an orange powder (113.2 mg, 82.5%). Anal. Calcd. For $C_{150}H_{132}N_{12}O_{12}$: C, 78.51; H, 5.80; N, 7.32. Found: C, 73.58; H, 5.59; N, 7.12.

Procedure for the preparation of COF-Ph

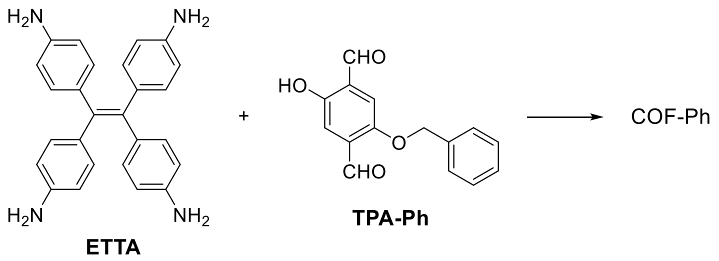

A mixture of ETTA (11.8 mg, 0.03 mmol), TPA-Ph (15.4 mg, 0.06 mmol), and *o*-dichlorobenzene (1 mL) in a glass ampoule was sonicated for 10 min and then acetic acid (aq., 6 M, 0.1 mL) was added. The ampoule was sealed after being degassed in a liquid nitrogen bath for 5 min, warmed to room temperature and then kept at 120 ^{o}C without disturbance for 3 days to yield a red solid. After being cooled to room temperature, the solvent was decanted and the solid was washed with dichloromethane and acetone for 3 times and then dried under dynamic vacuum at 120 ^{o}C for 2 h to

afford a red powder (17.9 mg, 70.5%). Anal. Calcd. For $C_{168}H_{120}N_{12}O_{12}$: C, 80.77; H, 4.81; N, 6.73. Found: C, 78.60; H, 4.85; N, 6.29.

Procedure for the preparation of COF-Na

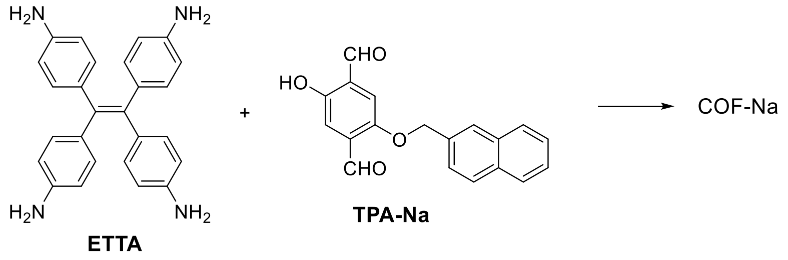

A mixture of ETTA (9.8 mg, 0.025 mmol), TPA-Na (15.3 mg, 0.05 mmol), and *o*-dichlorobenzene (1 mL) in a glass ampoule was sonicated for 10 min and then acetic acid (aq., 3 M, 0.1 mL) was added. The ampoule was sealed after being degassed in a liquid nitrogen bath for 5 min, warmed to room temperature and then kept at 120 ^{o}C without disturbance for 3 days to yield a red solid. After being cooled to room temperature, the solvent was decanted and the solid was washed with dichloromethane and acetone for 3 times and then dried under dynamic vacuum at 120 ^{o}C for 2 h to afford a red powder (15.5 mg, 66.5%). Anal. Calcd. For $C_{192}H_{132}N_{12}O_{12}$: C, 82.40; H, 4.72; N, 6.01. Found: C, 77.99; H, 4.58; N, 6.09.

Procedure for the preparation of SIOC-COF[2]

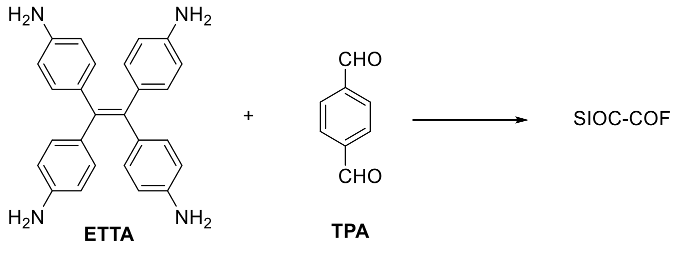

A mixture of 4,4',4'',4'''-(ethene-1,1,2,2-tetrayl)tetraaniline (ETTA, 30 mg, 0.076 mmol), terephthalaldehyde (TPA, 20.5 mg, 0.153 mmol), and 1,4-dioxane (1 mL) in a glass ampoule was sonicated for 10 min and then acetic acid (aq., 6 M, 0.1 mL) was added. The ampoule was sealed after being degassed in a liquid nitrogen bath for 5 min, warmed to room temperature and then kept at 120 ^{o}C without disturbance for 3 days to

yield a yellow solid. After being cooled to room temperature, the solvent was decanted and the solid was washed with THF and acetone for 3 times and then dried under dynamic vacuum at 120 °C for 2 h to afford a yellow powder (40.6 mg, 90.2%).

Procedure for the preparation of COF-TAB-Hex

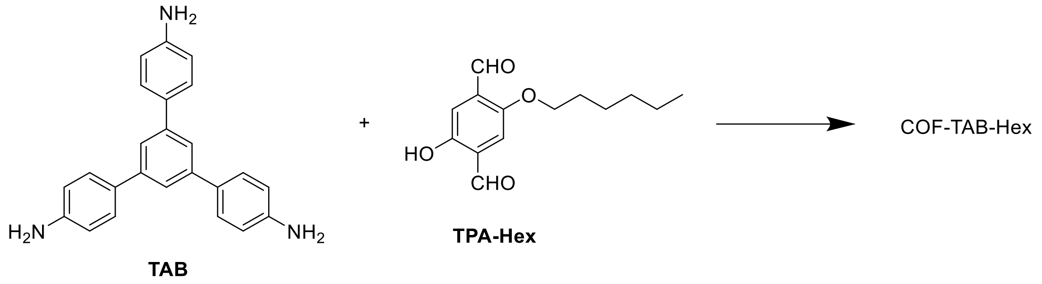

A mixture of 1,3,5-tri-(4-aminophenyl) benzene (TAP, 17.6 mg, 0.051 mmol), 2-hexyloxy-5-hydroxyterephthalaldehyde (TPA-Hex, 20.0 mg, 0.076 mmol), o-dichlorobenzene (*o*-DCB, 0.5 ml), and *n*-BuOH (0.5 ml) in a glass ampoule was sonicated for 10 min and then acetic acid (aq., 6 M, 0.1 mL) was added. The ampoule was sealed after being degassed in a liquid nitrogen bath for 5 min, warmed to room temperature and then kept at 120 °C without disturbance for 3 days to yield yellow solid. After being cooled to room temperature, the solvent was decanted and the solid was washed with THF and acetone for 3 times and then dried under dynamic vacuum at 120 °C for 2 h to afford a yellow powder (27.5 mg, 81.6%). Anal. Calcd. For $C_{90}H_{84}N_6O_6$: C, 80.33; H, 6.29; N, 6.25. Found: C, 78.96; H, 6.23; N, 6.23.

Section C. Characterization of COF-Bu, COF-Ph and COF-Na.

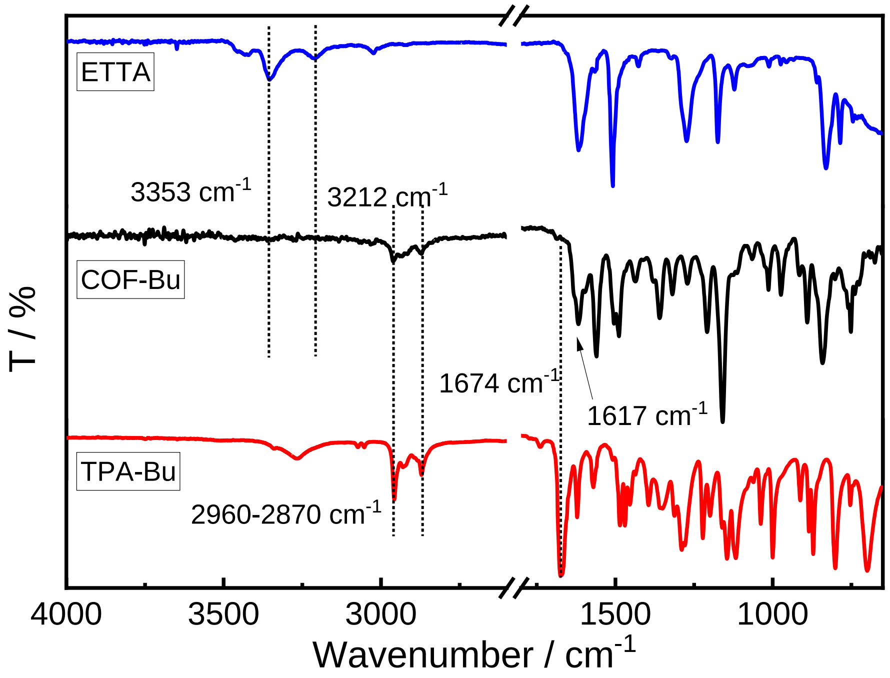

Supplementary Fig. 1 FT-IR spectra of ETTA, COF-Bu, and TPA-Bu.

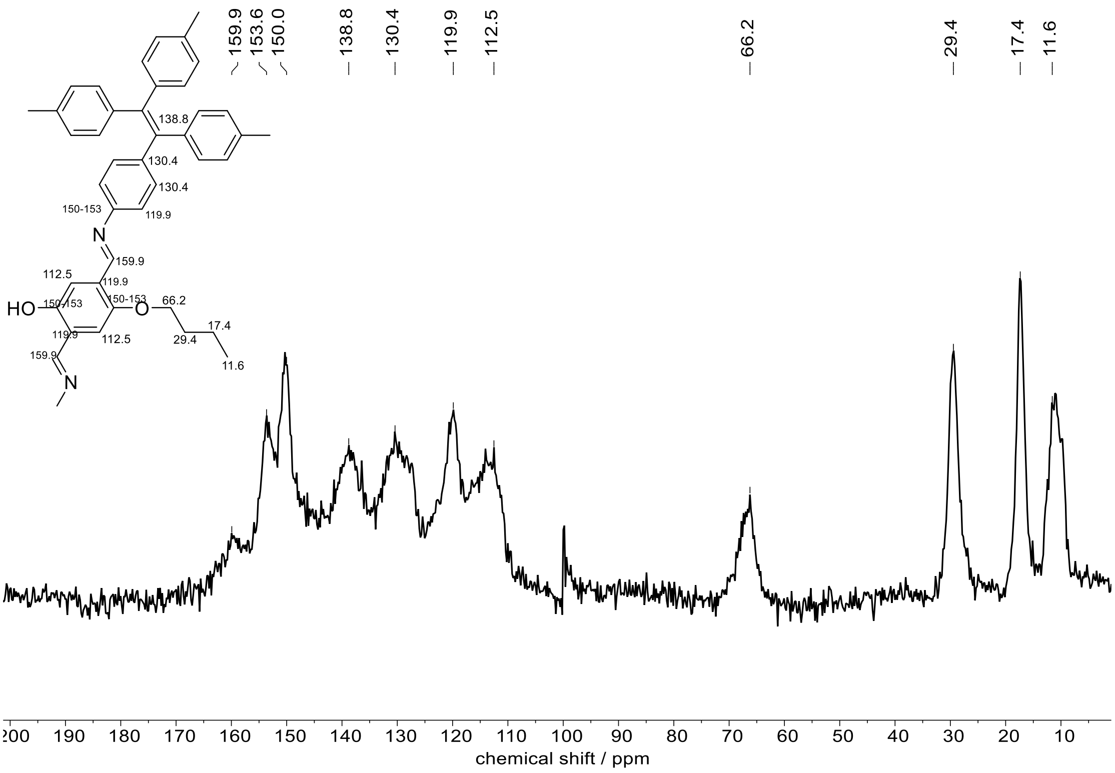

Supplementary Fig. 2 Solid-state ^{13}C CP/MAS NMR spectrum of COF-Bu.

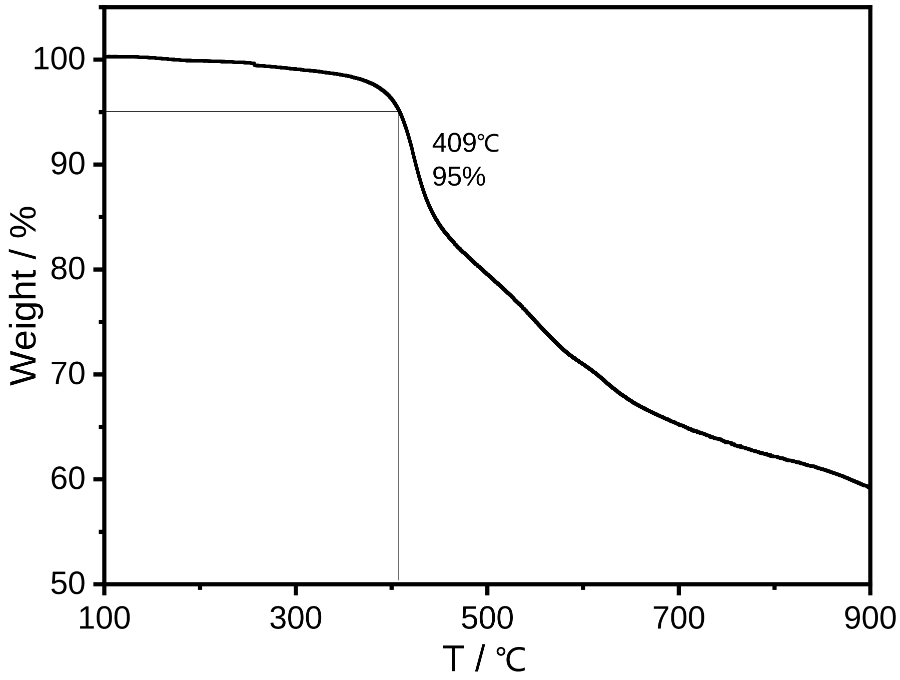

Supplementary Fig. 3 TGA profile of COF-Bu.

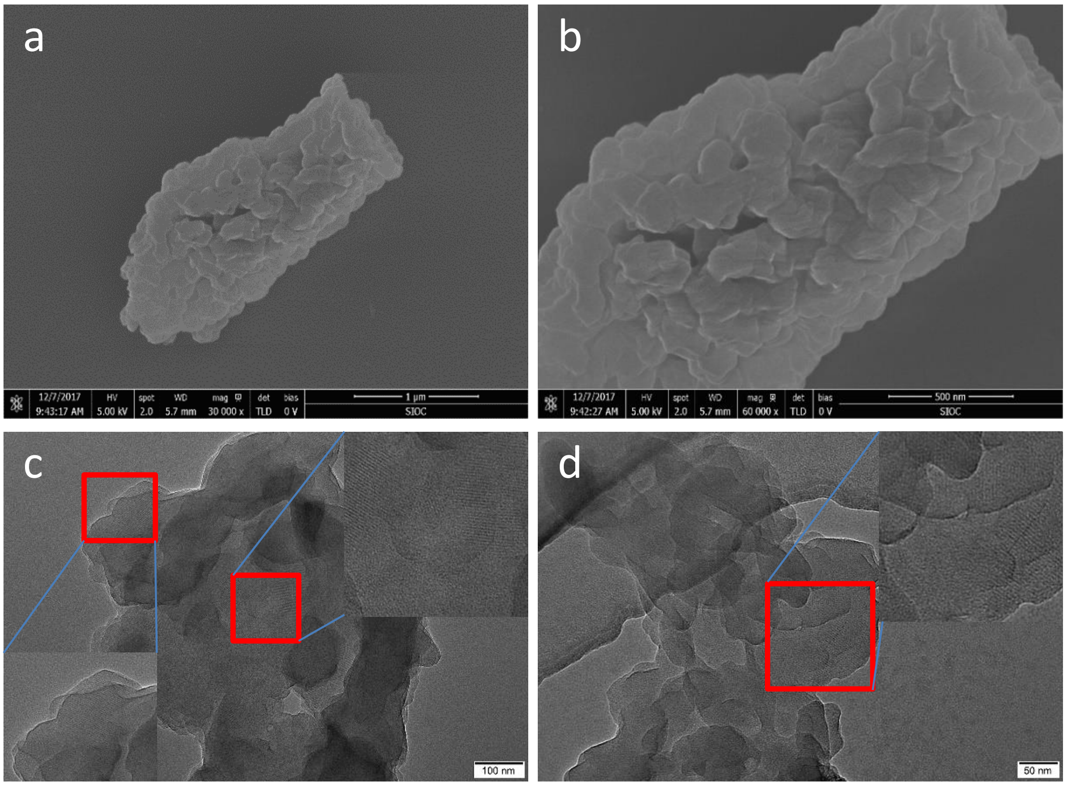

Supplementary Fig. 4 (a-b) SEM images and (c-d) TEM images of COF-Bu.

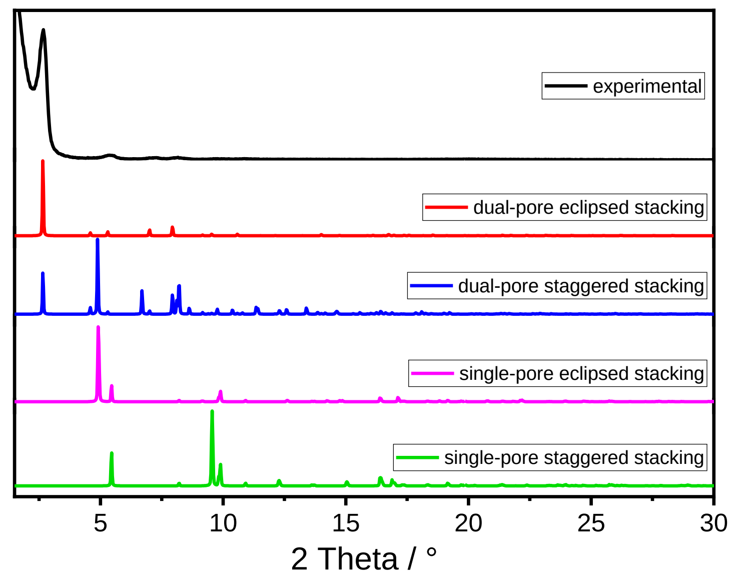

Supplementary Fig. 5 Experimental and simulated PXRD patterns for the possible structures of COF-Bu based on dual-pore and single-pore frameworks with eclipsed and staggered stacking, respectively.

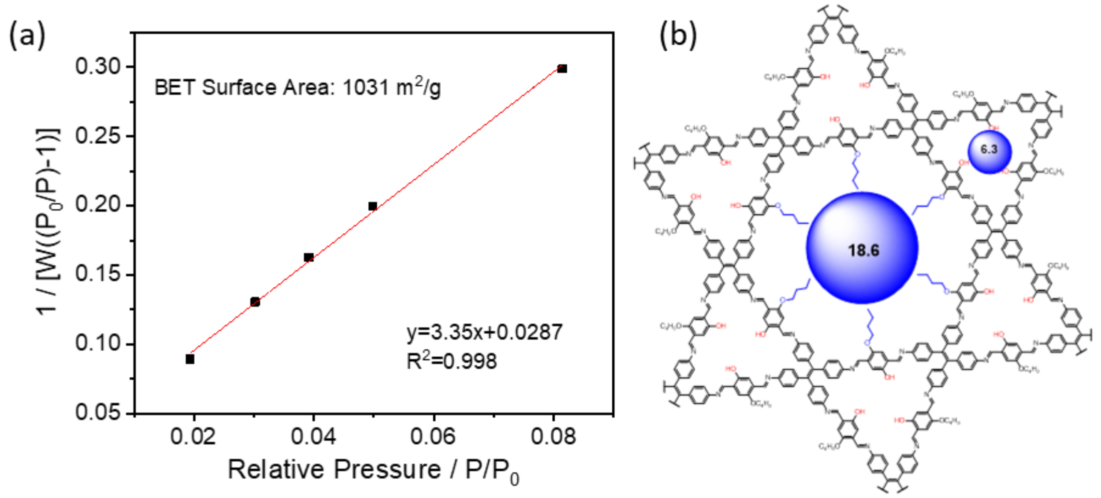

Supplementary Fig. 6 (a) BET surface area plot of COF-Bu. (b) Schematic diagram of theoretical pore size of COF-Bu.

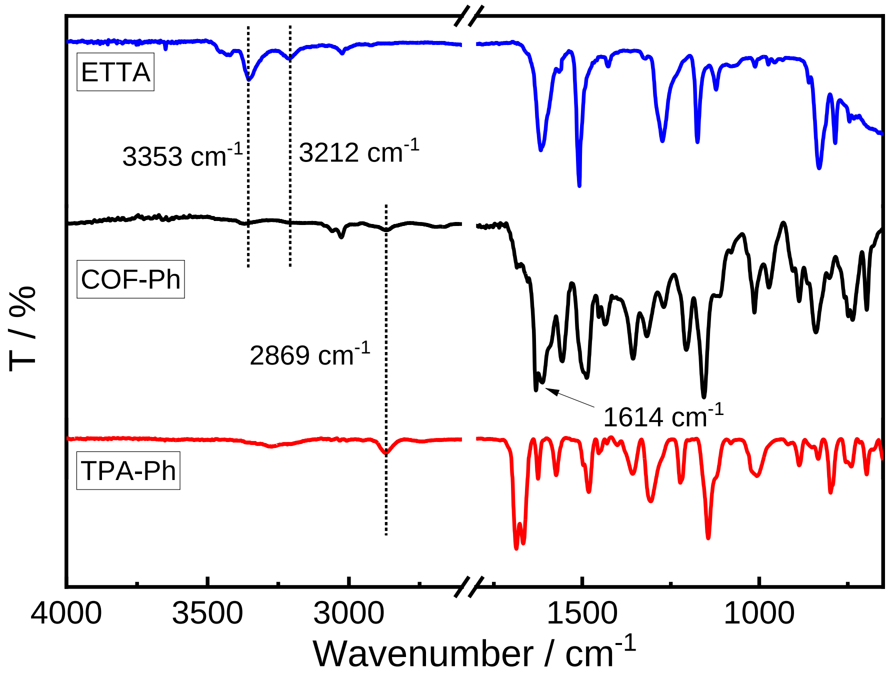

Supplementary Fig. 7 FT-IR spectra of ETTA, COF-Ph, and TPA-Ph.

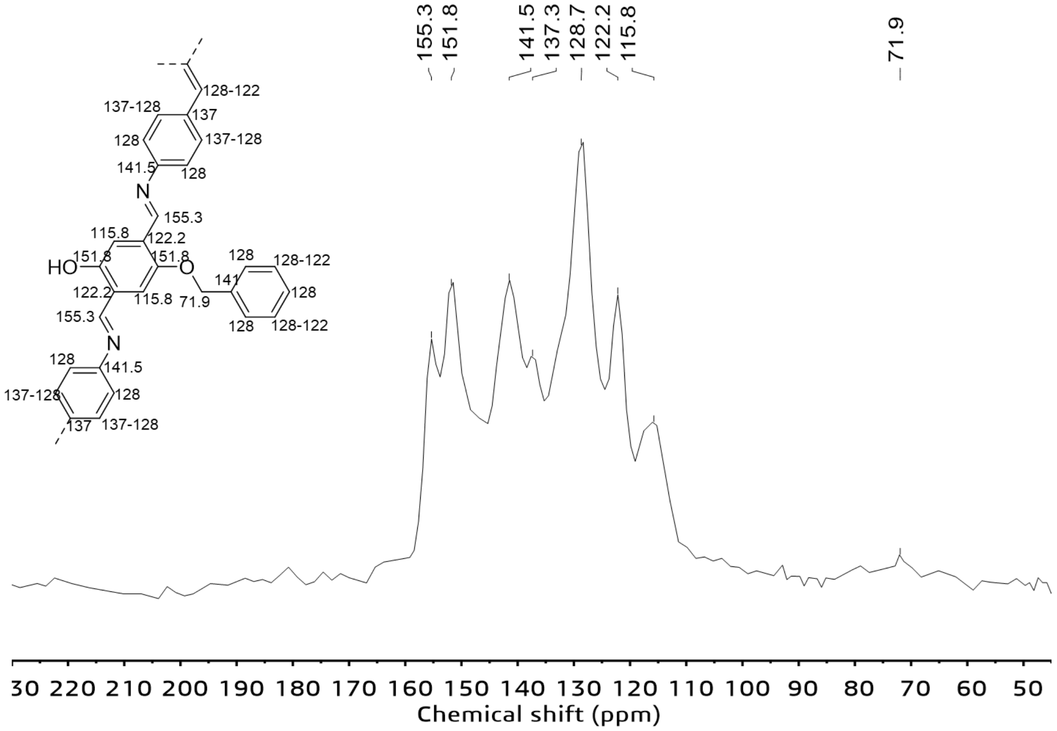

Supplementary Fig. 8 Solid-state ^{13}C CP/MAS NMR spectrum of COF-Ph.

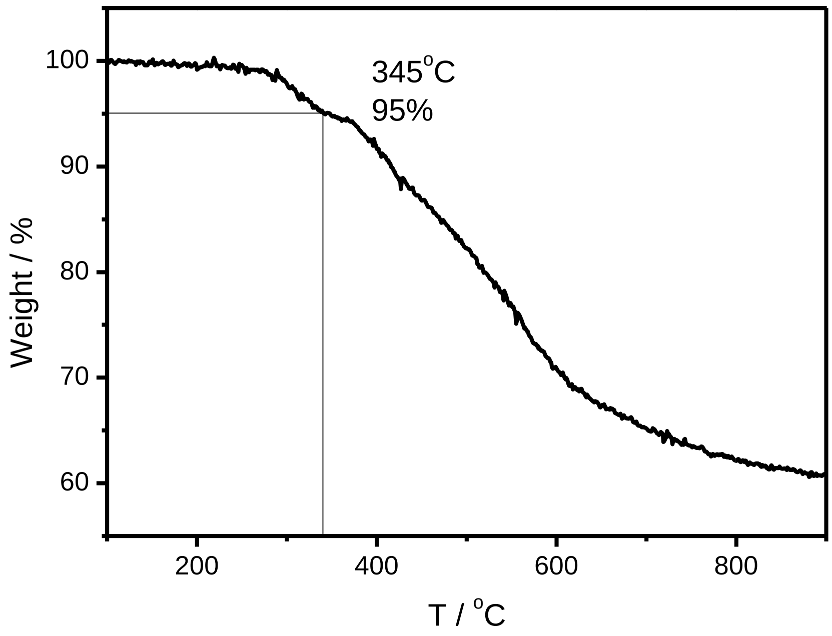

Supplementary Fig. 9 TGA profile of COF-Ph.

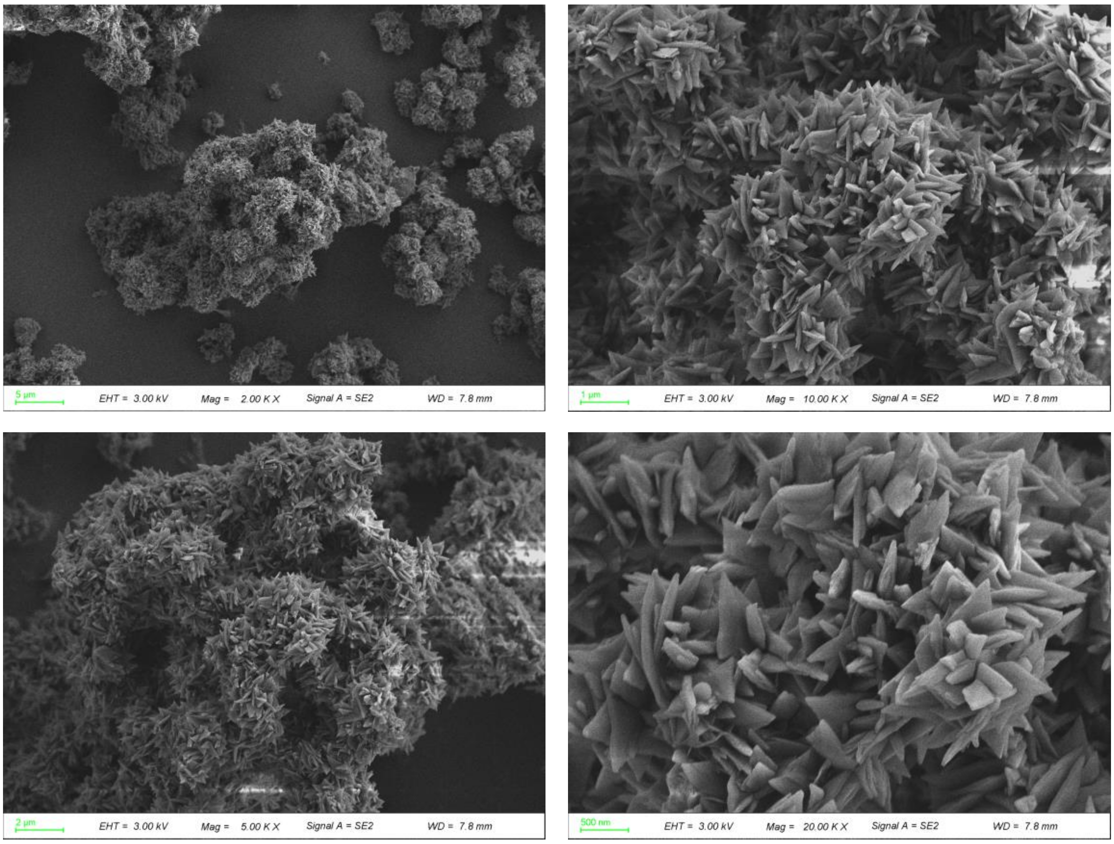

Supplementary Fig. 10 SEM images of COF-Ph.

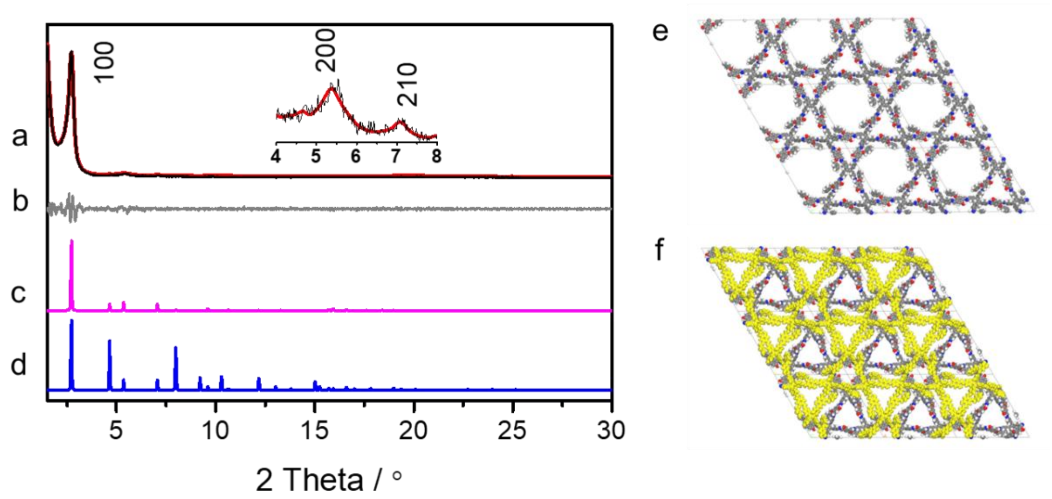

Supplementary Fig. 11 (a) Experimental (black) and refined (red) PXRD patterns of COF-Ph. (b) Difference plot between the experimental and refined PXRD patterns. Simulated PXRD patterns for (c) eclipsed and (d) staggered dual-pore structures. Structural representation of COF-Ph with (e) eclipsed and (f) staggered stacking.

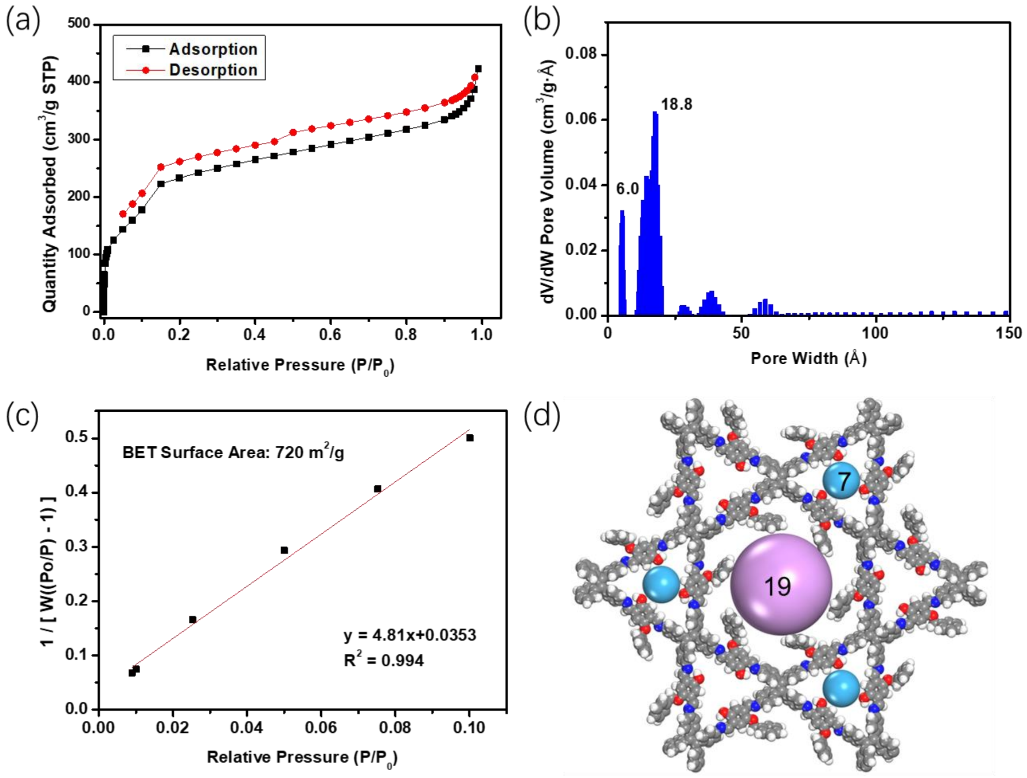

Supplementary Fig. 12 (a) N_2 adsorption-desorption isotherm at 77 K, (b) pore size distribution profile, (c) BET surface area plot, and (d) schematic diagram of theoretical pore size of COF-Ph.

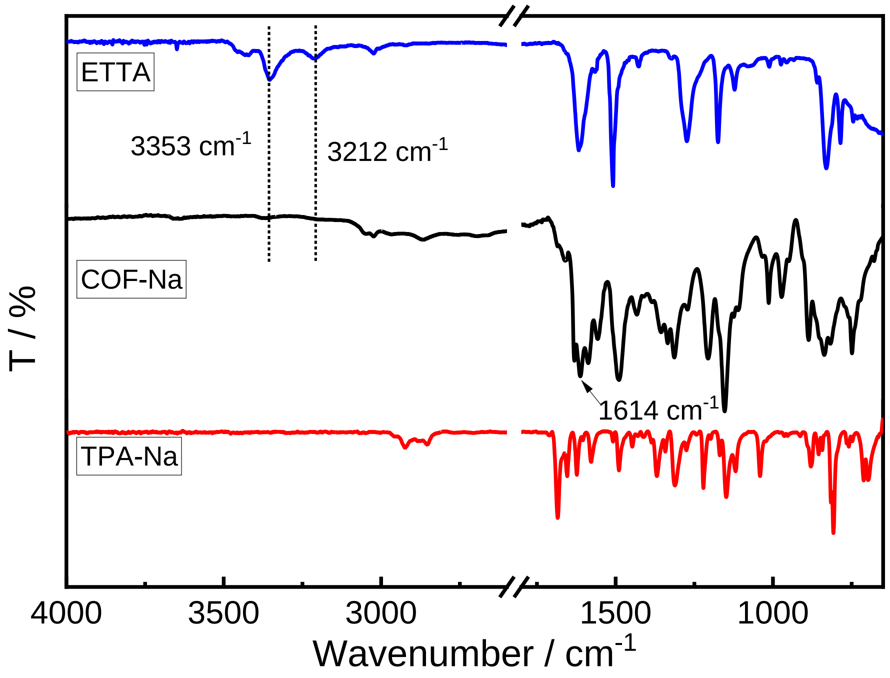

Supplementary Fig. 13 FT-IR spectra of ETTA, COF-Na, and TPA-Na.

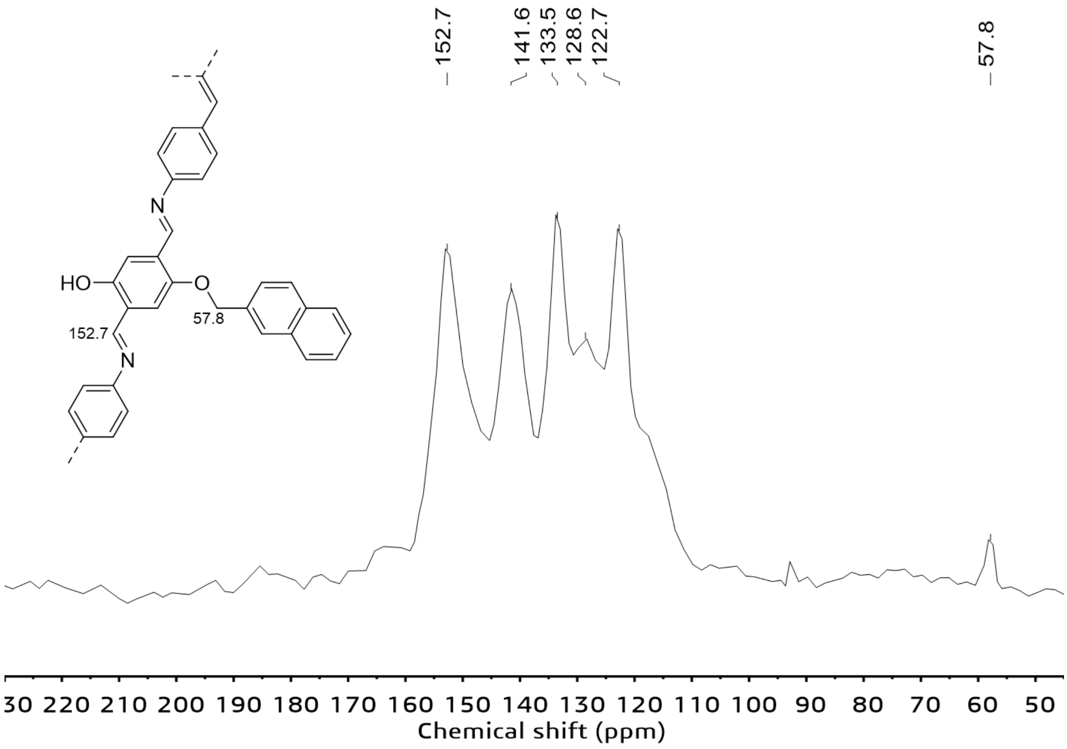

Supplementary Fig. 14 Solid-state ^{13}C CP/MAS NMR spectrum of COF-Na.

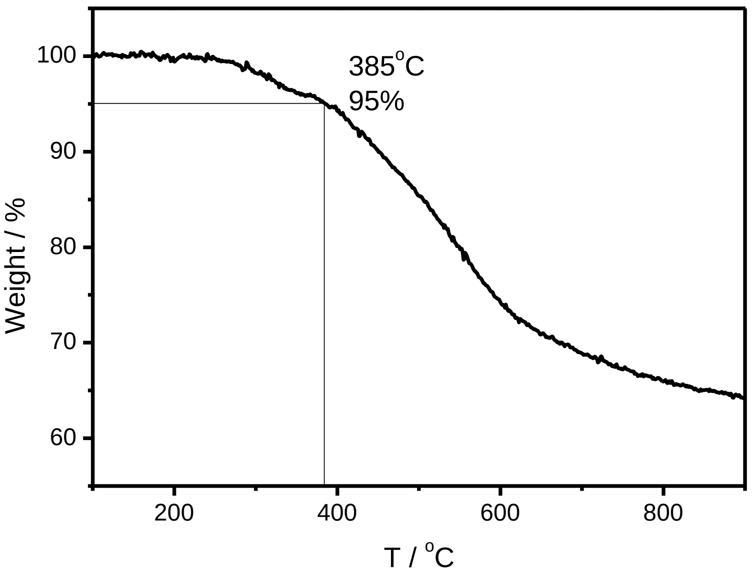

Supplementary Fig. 15 TGA profile of COF-Na.

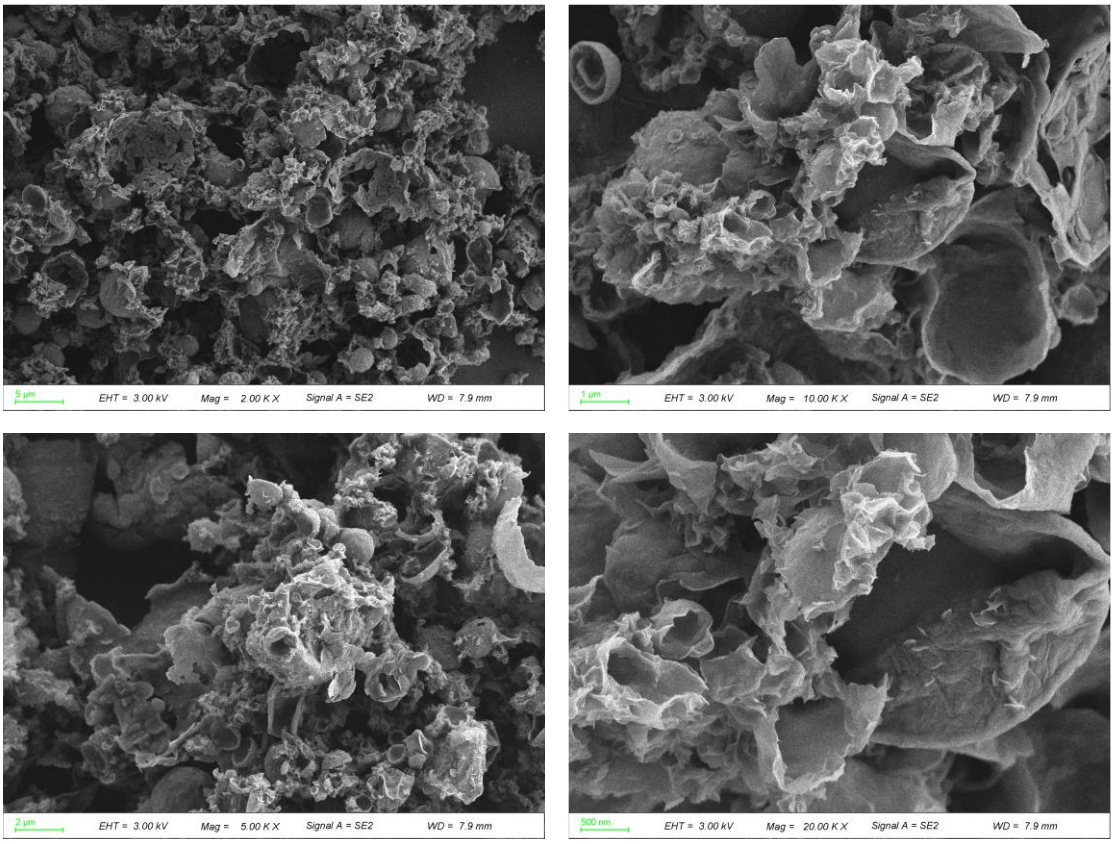

Supplementary Fig. 16 SEM images of COF-Na.

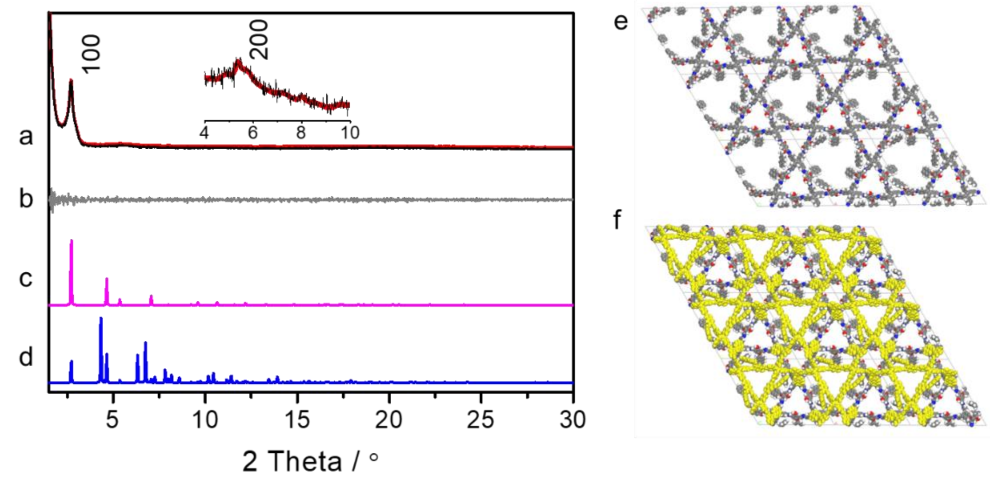

Supplementary Fig. 17 (a) Experimental (black) and refined (red) PXRD patterns of COF-Na. (b) Difference plot between the experimental and refined PXRD patterns. Simulated PXRD patterns for (c) eclipsed and (d) staggered dual-pore structures. Structural representation of COF-Na with (e) eclipsed and (f) staggered stacking.

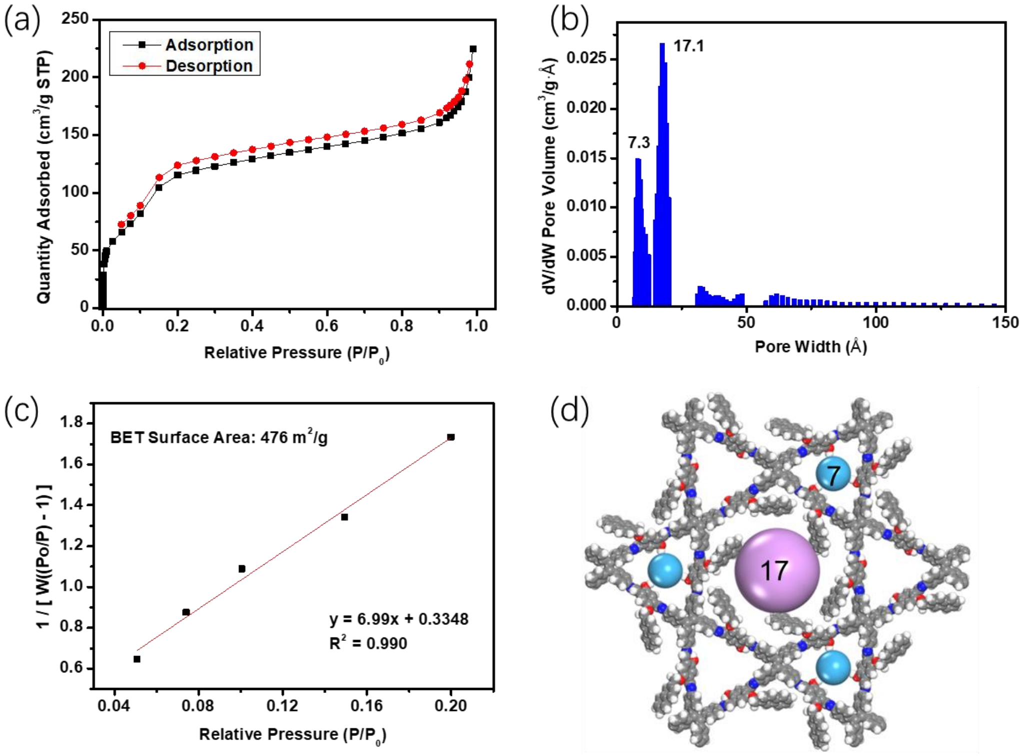

Supplementary Fig. 18 (a) N_2 adsorption-desorption isotherm at 77 K, (b) pore size distribution profile, (c) BET surface area plot, and (d) schematic diagram of theoretical pore size of COF-Na.

Section D. Characterization of the COF-Bu and COF-Ph membranes.

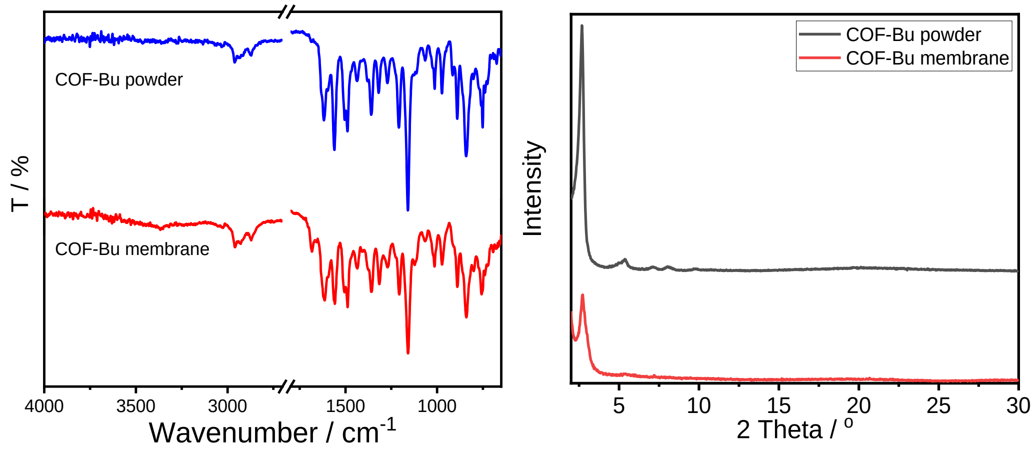

Supplementary Fig. 19 Comparison of FT-IR spectra and PXRD patterns between the powder and membrane of COF-Bu.

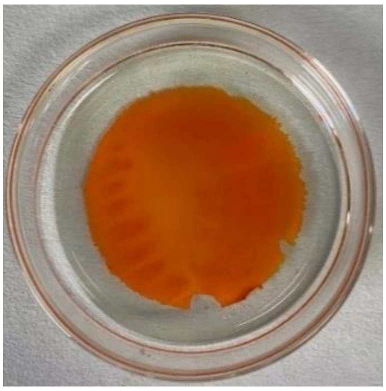

Supplementary Fig. 20 Photograph of the COF-Bu membrane.

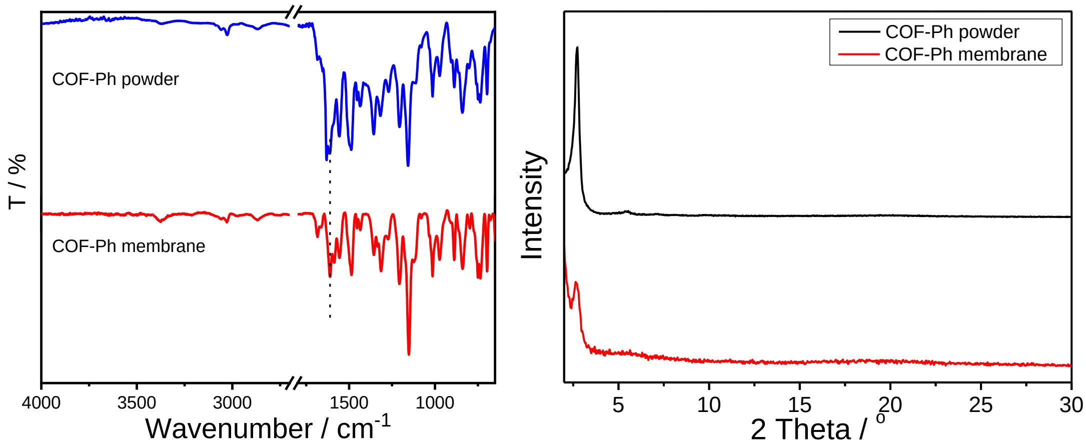

Supplementary Fig. 21 Comparison of FT-IR spectra and PXRD patterns between the powder and membrane of COF-Ph.

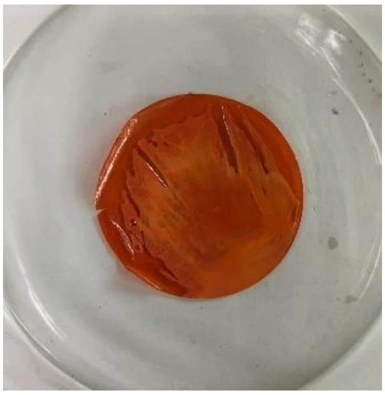

Supplementary Fig. 22 Photograph of the COF-Ph membrane.

Section E. Characterization of COF-TAB-Hex and SIOC-COF.

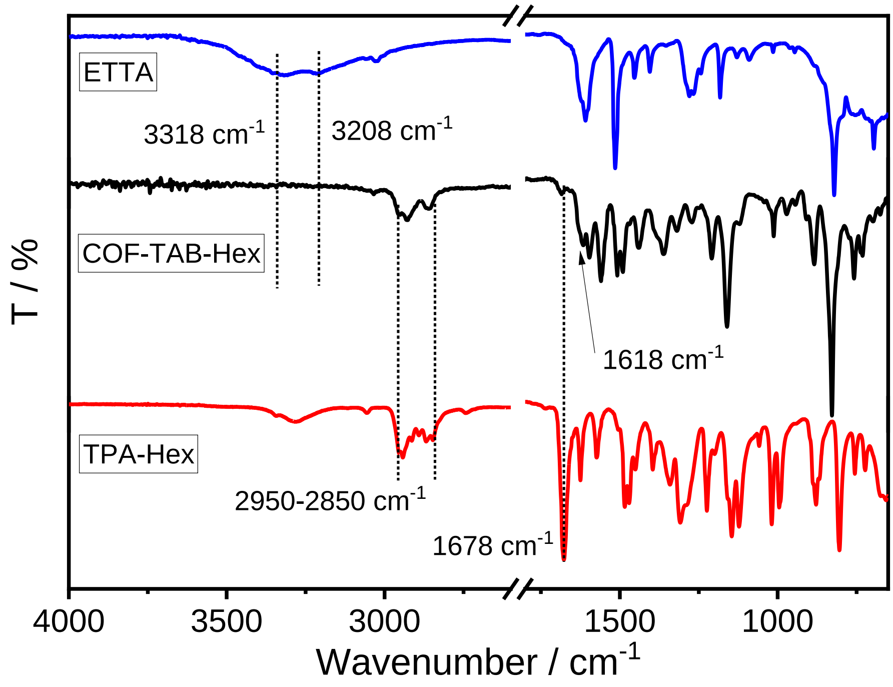

Supplementary Fig. 23 FT-IR spectra of COF-TAB-Hex and its monomers.

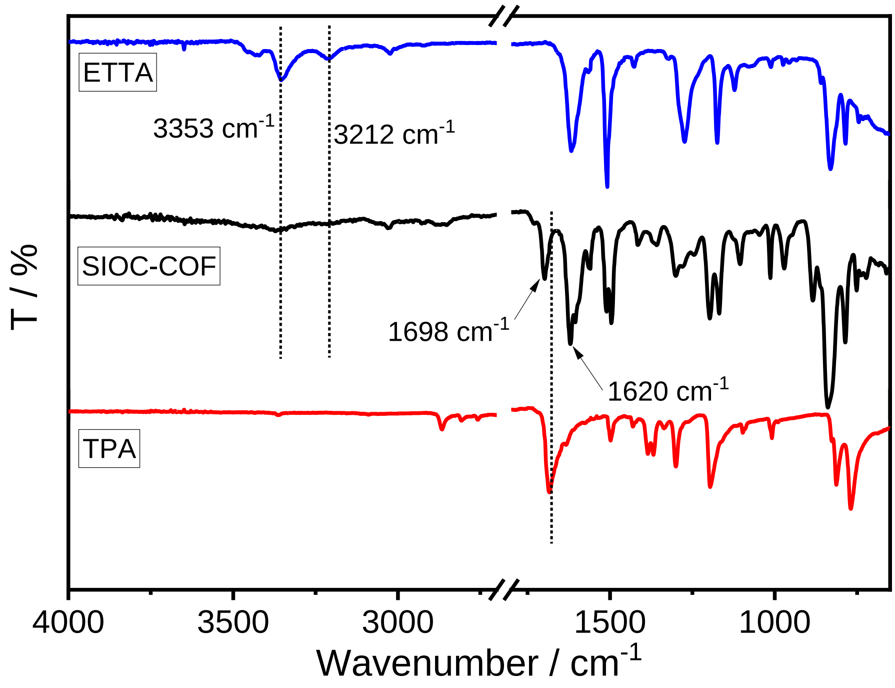

Supplementary Fig. 24 FT-IR spectra of SIOC-COF and its monomers.

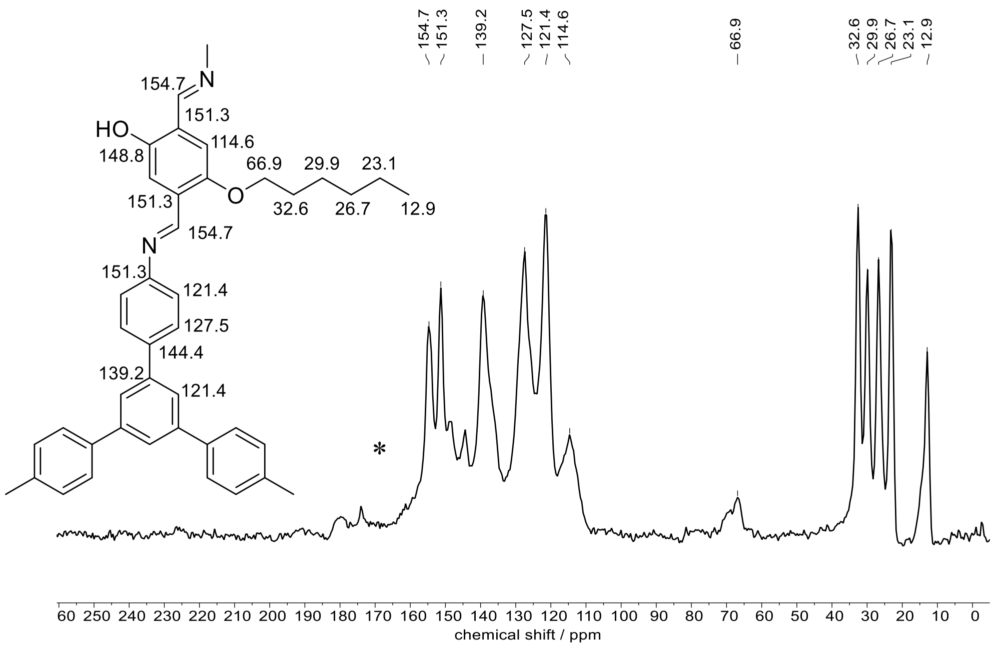

Supplementary Fig. 25 Solid-state ^{13}C CP/MAS NMR spectrum of COF-TAB-Hex. The signal marked with * is a side band.

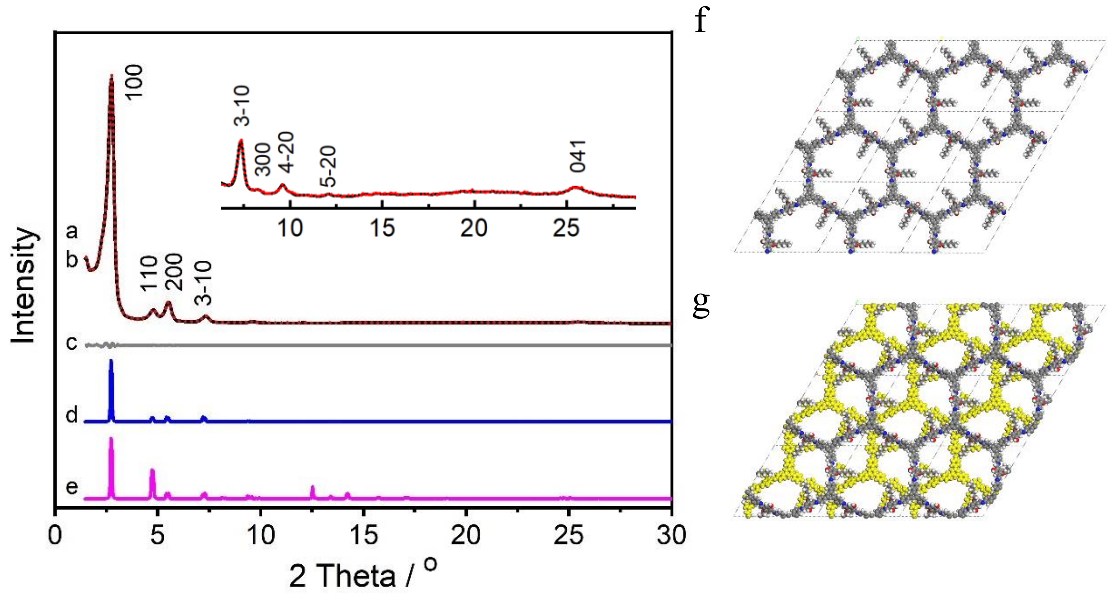

Supplementary Fig. 26 (a) Experimental (black line) and (b) refined (red dot line) PXRD patterns of COF-TAB-Hex. (c) Difference plot between the experimental and refined PXRD patterns. Simulated PXRD patterns for COF-TAB-Hex with (d) eclipsed and (e) staggered stacking. Structural representation of COF-TAB-Hex with (f) eclipsed stacking and (g) staggered stacking.

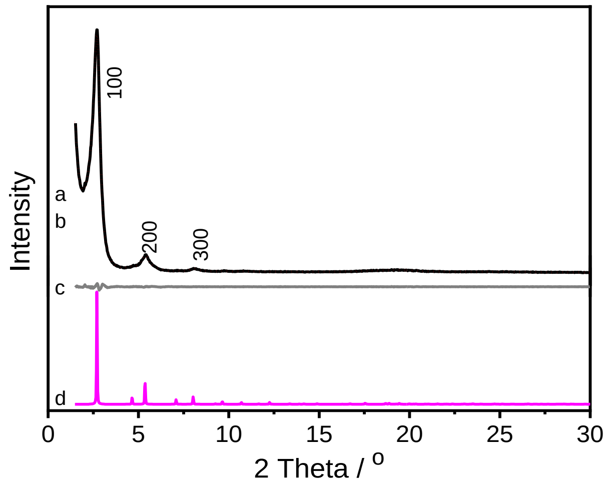

Supplementary Fig. 27 (a) Experimental (black line) and (b) refined (red dot line) PXRD patterns of SIOC-COF. (c) Difference plot between the experimental and refined PXRD patterns. (d) Simulated PXRD pattern of SIOC-COF with eclipsed stacking.

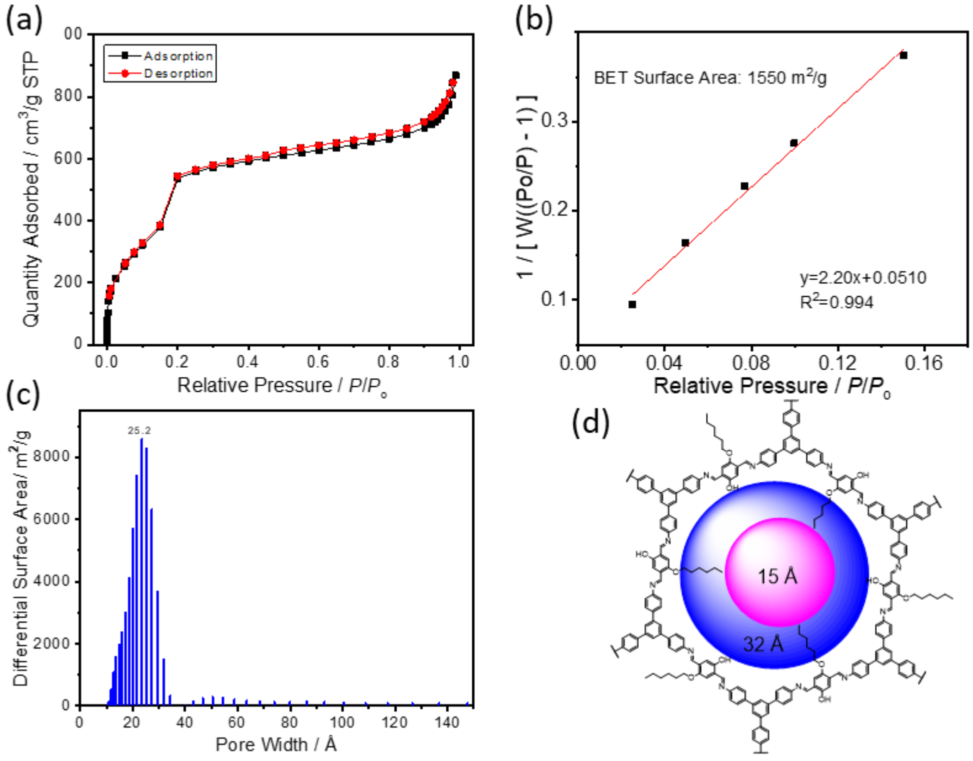

Supplementary Fig. 28 (a) N_2 adsorption-desorption isotherm; (b) BET surface area plot; (c) pore size distribution profile, and (d) schematic diagram of theoretical pore size of COF-TAB-Hex. Total pore volume (at $P/P_0 = 0.99$) is 1.34 cm^3/g.

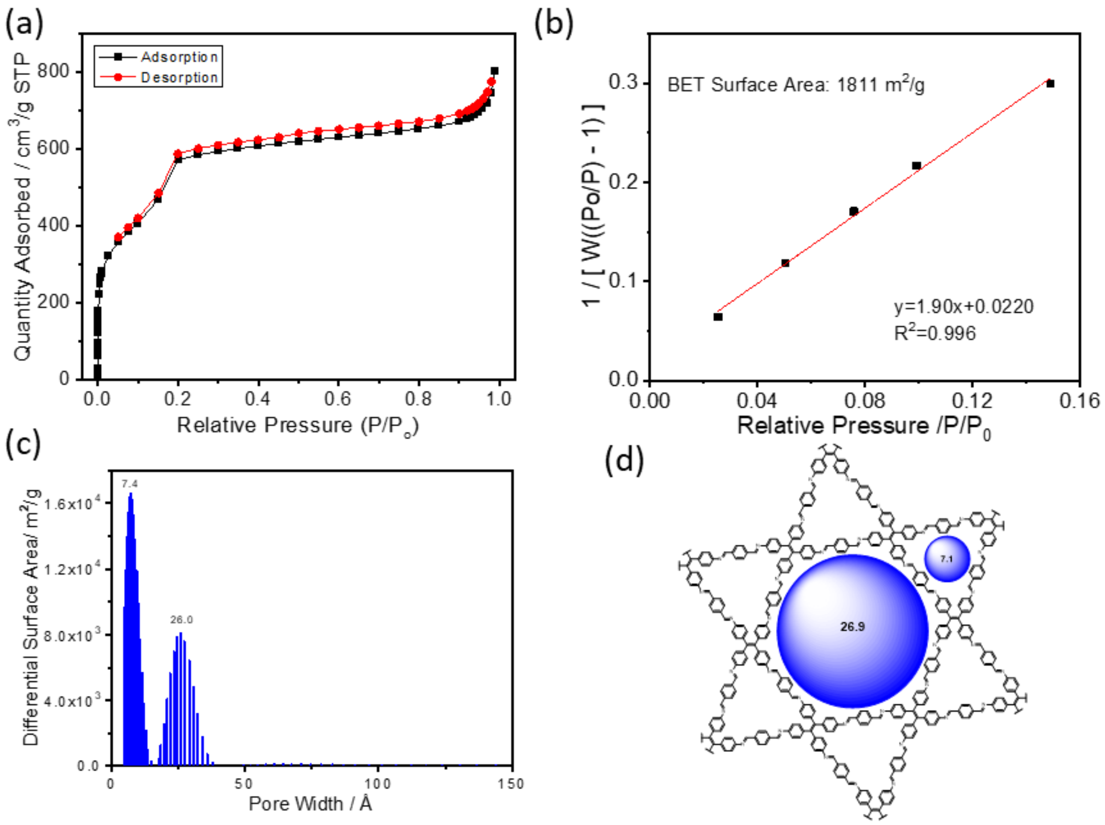

Supplementary Fig. 29 (a) N_2 adsorption-desroption isotherm; (b) BET surface area plot; (c) pore size distribution profile, and (d) schematic diagram of theoretical pore size of SIOC-COF. Total pore volume (at $P/P_0 = 0.99$) is 1.24 cm^3/g.

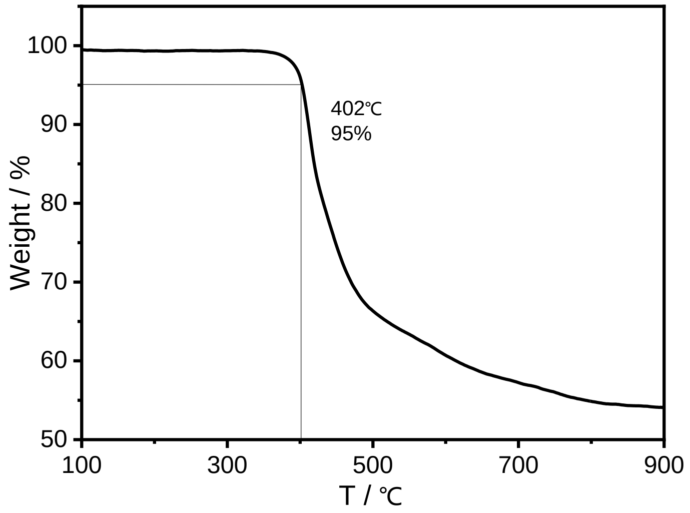

Supplementary Fig. 30 TGA profile of COF-TAB-Hex.

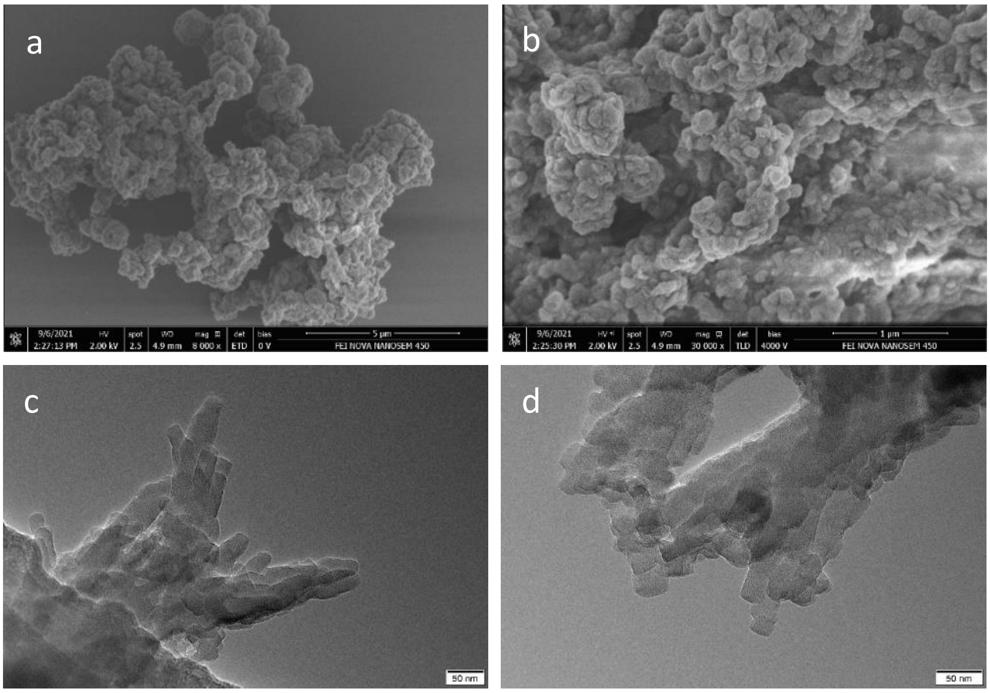

Supplementary Fig. 31 (a-b) SEM images and (c-d) TEM images of COF-TAB-Hex.

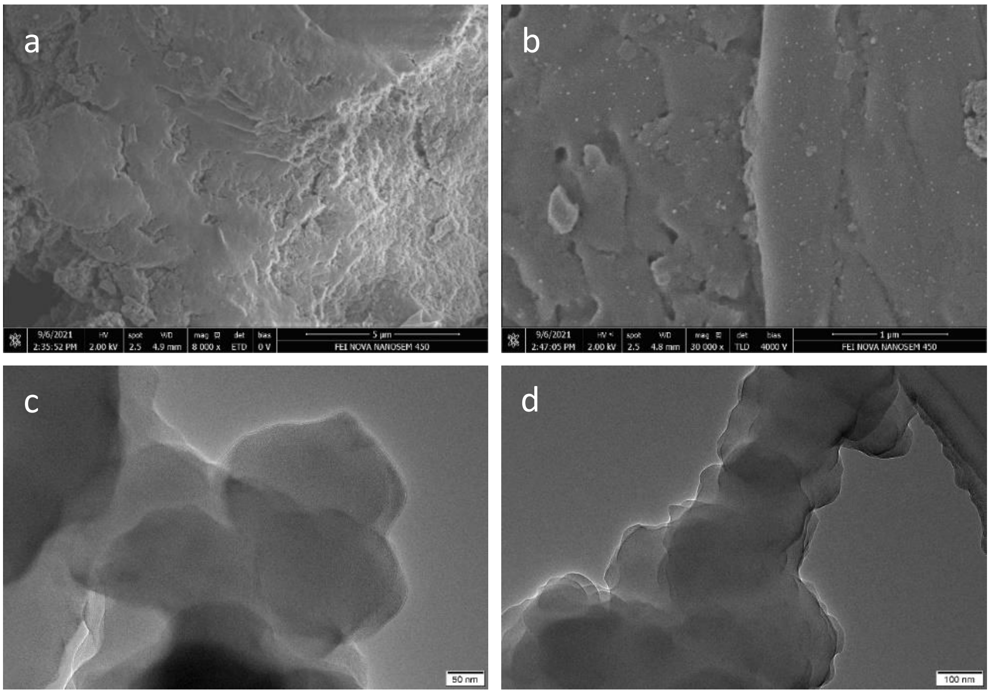

Supplementary Fig. 32 (a-b) SEM images and (c-d) TEM images of SIOC-COF.

Section F. Characterization of the COF-TAB-Hex, SIOC-COF, and POP-Bu membranes.

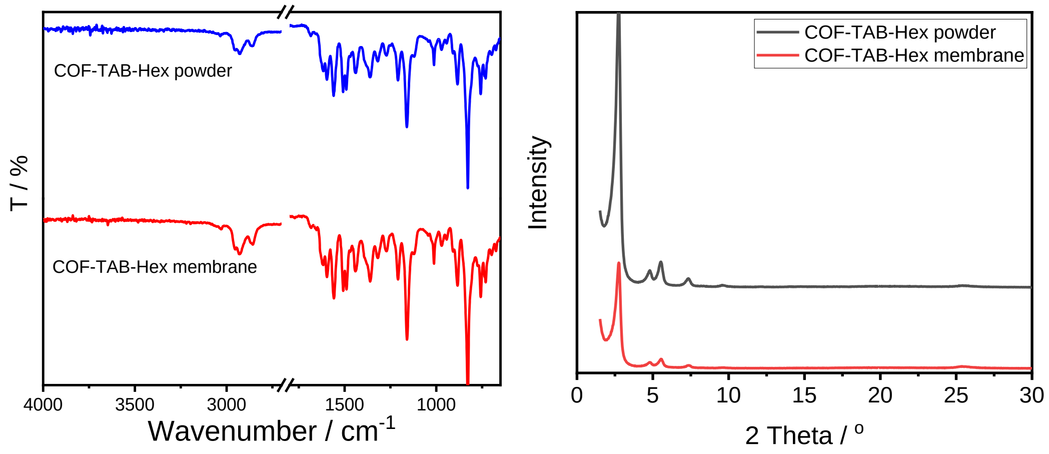

Supplementary Fig. 33 Comparision between FT-IR spectra and PXRD patterns of the powder and membrane of COF-TAB-Hex.

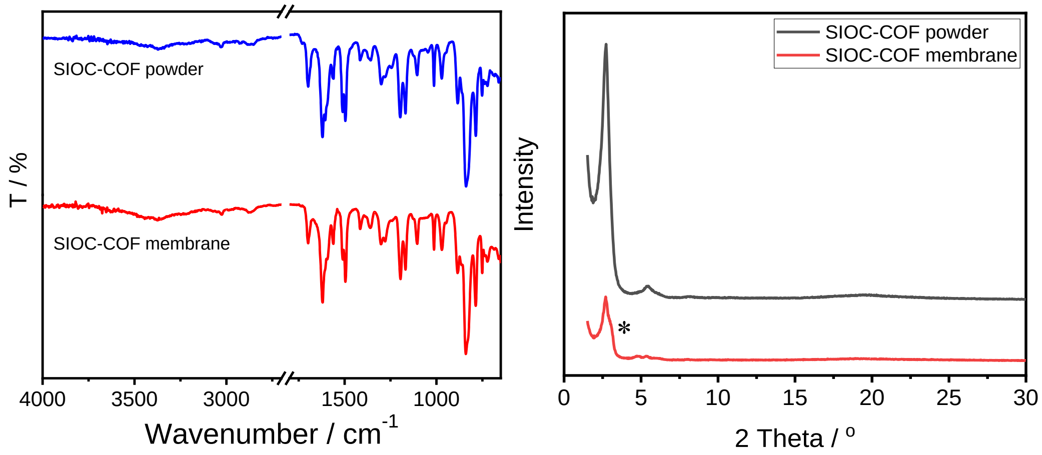

Supplementary Fig. 34 Comparison between FT-IR spectra and PXRD patterns of the powder and membrane of SIOC-COF.

Note: The small shoulder on the main diffraction peak of the SIOC-COF membrane marked with * could probably be attributed to a partial staircase-like stacking of the COF layers synthesized by interfacial-polymerization.[3,4]

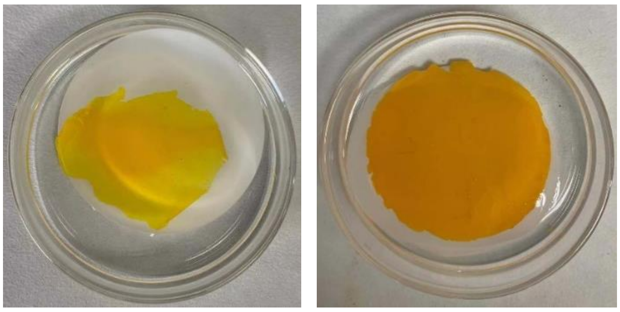

Supplementary Fig. 35 Photographs of the SIOC-COF membrane (left) and the COF-TAB-Hex membrane (right).

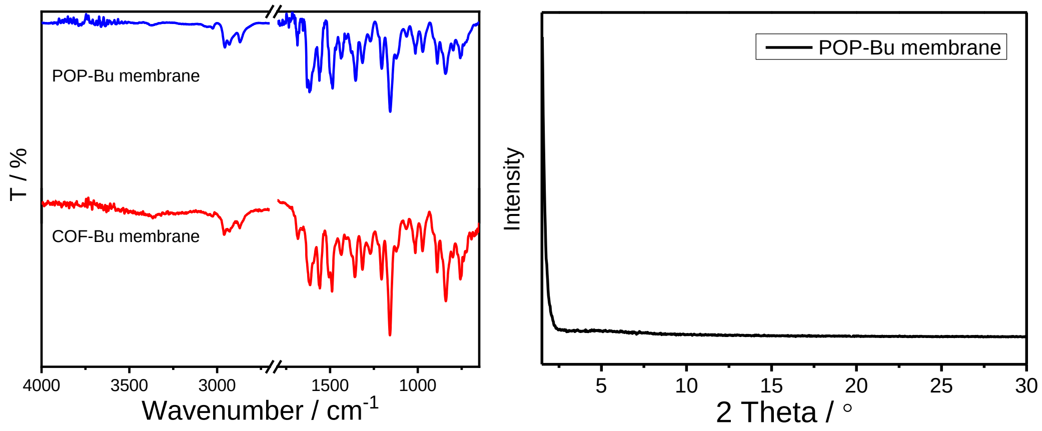

Supplementary Fig. 36 Left: Comparison between FT-IR spectra of the POP-Bu and COF-Bu membranes; Right: PXRD pattern of the POP-Bu membrane.

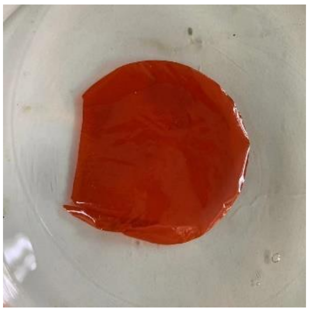

Supplementary Fig. 37 Photograph of the POP-Bu membrane.

Section G. SEM and contact angle images of the membranes.

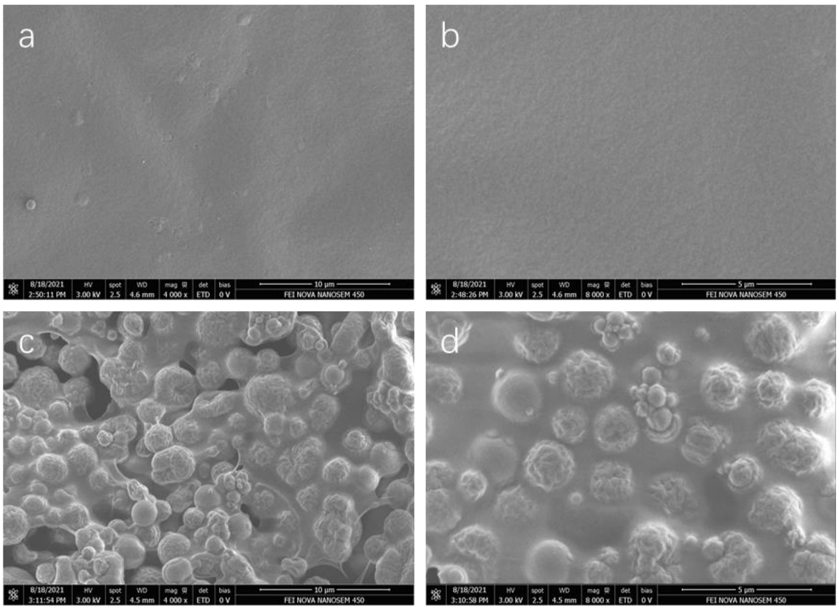

Supplementary Fig. 38 SEM images of the COF-Bu membrane under different resolution. (a-b) upper surface, and (c-d) bottom surface.

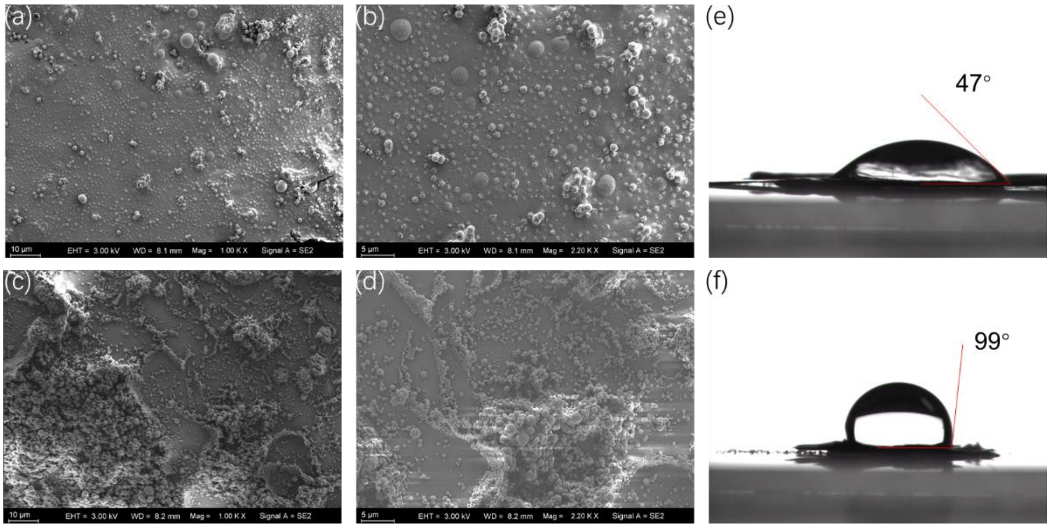

Supplementary Fig. 39 SEM images: (a-b) upper surface and (c-d) bottom surface of the COF-Ph membrane; Contact angles: (e) upper surface and (f) bottom surface of the COF-Ph membrane.

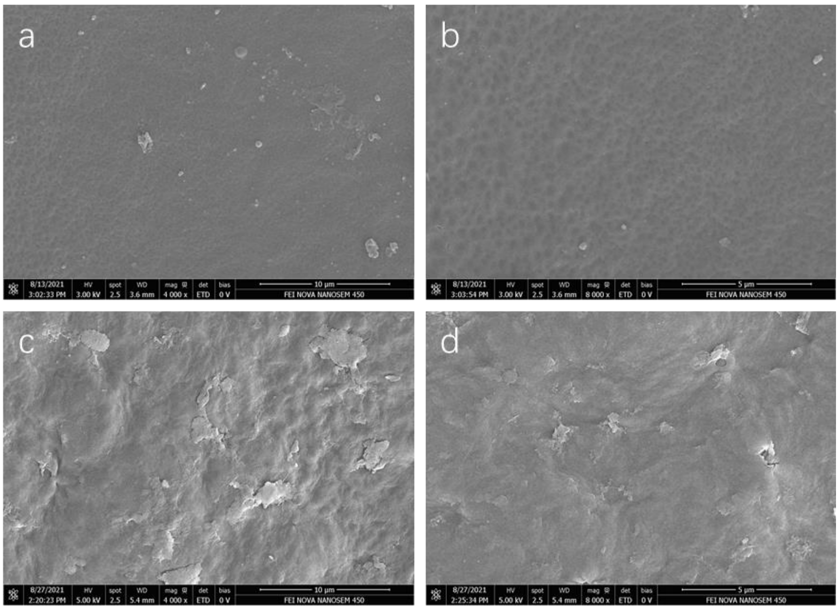

Supplementary Fig. 40 SEM images of the SIOC-COF membrane under different resolution. (a-b) upper surface, and (c-d) bottom surface.

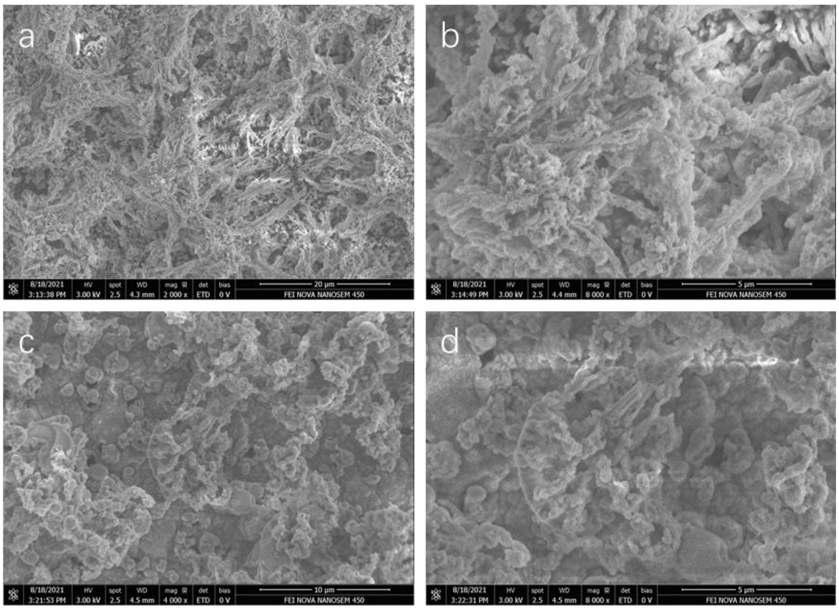

Supplementary Fig. 41 SEM images of the COF-TAB-Hex membrane under different resolution. (a-b) upper surface, and (c-d) bottom surface.

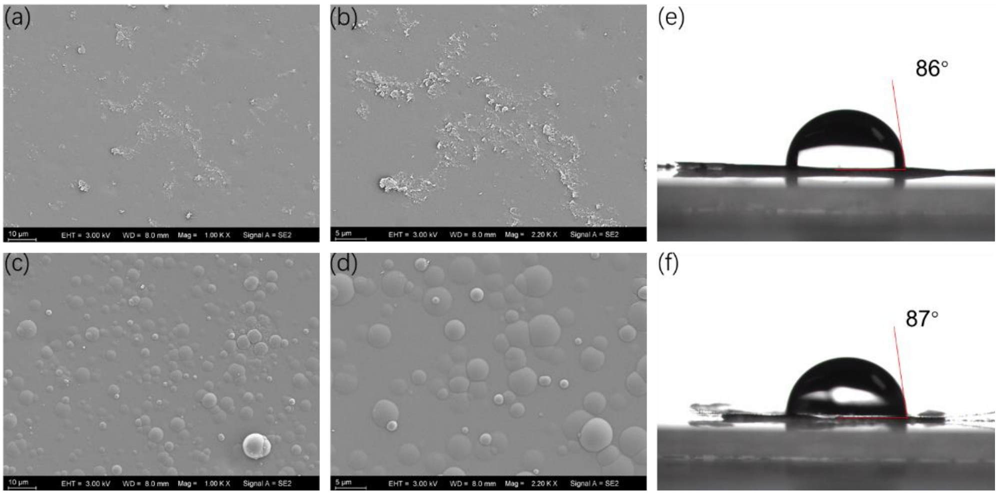

Supplementary Fig. 42 SEM images: (a-b) upper surface and (c-d) bottom surface of the POP-Bu membrane; Contact angles: (e) upper surface and (f) bottom surface of the POP-Bu membrane.

Section H. Fractional atomic coordinates of the COFs.

Supplementary Table 1. Fractional atomic coordinates for the unit cell of COF-Bu with eclipsed stacking.

P3							
$a = b = 38.7523$, $c = 5.3075$; $\alpha = \beta = 90°$, $\gamma = 120°$							
C1	0.51732	0.99931	0.60459	H37	0.5535	0.97553	0.94302
C2	0.55739	0.03575	0.61156	H38	0.65453	0.17827	0.62944
C3	0.51733	0.96108	0.60722	H39	0.85673	0.30518	0.91667
C4	0.58766	0.03924	0.44918	H40	0.73484	0.17785	0.96991
C5	0.62593	0.07244	0.46323	H41	0.77989	0.29943	0.453
C6	0.63412	0.1028	0.63618	H42	0.71793	0.2682	0.26173
C7	0.60425	0.09919	0.80083	H43	0.79182	0.17643	1.10093
C8	0.56643	0.0657	0.79014	H44	0.77327	0.19674	1.33953
C9	0.49734	0.93232	0.42142	H45	0.84064	0.23487	1.52424
C10	0.49677	0.89612	0.42837	H46	0.86067	0.21748	1.28195
C11	0.51655	0.8882	0.62027	H47	0.81624	0.14681	1.42448
C12	0.53734	0.91737	0.80574	H48	0.79596	0.16407	1.66667
C13	0.53781	0.95359	0.79772	H49	0.88337	0.18398	1.61374
N14	0.67319	0.13675	0.64892	H50	0.84768	0.15251	1.84826
N15	0.84995	0.33611	0.62046	H51	0.86571	0.20541	1.84763
C16	0.67915	0.17275	0.65455	C52	0.48268	0.00069	0.60459
C17	0.83752	0.30595	0.77212	C53	0.44261	0.96425	0.61156
C18	0.71954	0.20662	0.68343	C54	0.48267	0.03892	0.60722
C19	0.79679	0.27168	0.74516	C55	0.41234	0.96076	0.44918
C20	0.74554	0.20473	0.85933	C56	0.37407	0.92756	0.46323
C21	0.78432	0.23735	0.89439	C57	0.36588	0.8972	0.63618
C22	0.77049	0.27319	0.56856	C58	0.39575	0.90081	0.80083
C23	0.73187	0.24101	0.53861	C59	0.43357	0.9343	0.79014
O24	0.70592	0.24241	0.36091	C60	0.50266	0.06768	0.42142
O25	0.81152	0.23722	1.07128	C61	0.50323	0.10388	0.42837
C26	0.79946	0.20258	1.22292	C62	0.48345	0.1118	0.62027
C27	0.83372	0.20949	1.39661	C63	0.46266	0.08263	0.80574
C28	0.82294	0.17216	1.55216	C64	0.46219	0.04641	0.79772
C29	0.85711	0.17902	1.72627	N65	0.32681	0.86325	0.64892
H30	0.5817	0.01622	0.31243	N66	0.15005	0.66389	0.62046
H31	0.64898	0.07482	0.33708	C67	0.32085	0.82725	0.65455
H32	0.61049	0.12198	0.94037	C68	0.16248	0.69405	0.77212
H33	0.54418	0.06313	0.92071	C69	0.28046	0.79338	0.68343
H34	0.48179	0.93788	0.27429	C70	0.20321	0.72832	0.74516
H35	0.4807	0.87402	0.28423	C71	0.25446	0.79527	0.85933

H36	0.55278	0.91258	0.95889	C72	0.21568	0.76265	0.89439
C73	0.22951	0.72681	0.56856	H88	0.4465	0.02447	0.94302
C74	0.26813	0.75899	0.53861	H89	0.34547	0.82173	0.62944
O75	0.29408	0.75759	0.36091	H90	0.14327	0.69482	0.91667
O76	0.18848	0.76278	1.07128	H91	0.26516	0.82215	0.96991
C77	0.20054	0.79742	1.22292	H92	0.22011	0.70057	0.453
C78	0.16628	0.79051	1.39661	H93	0.28207	0.7318	0.26173
C79	0.17706	0.82784	1.55216	H94	0.20818	0.82357	1.10093
C80	0.14289	0.82098	1.72627	H95	0.22673	0.80326	1.33953
H81	0.4183	0.98378	0.31243	H96	0.15936	0.76513	1.52424
H82	0.35102	0.92518	0.33708	H97	0.13933	0.78252	1.28195
H83	0.38951	0.87802	0.94037	H98	0.18376	0.85319	1.42448
H84	0.45582	0.93687	0.92071	H99	0.20404	0.83593	1.66667
H85	0.51821	0.06212	0.27429	H100	0.11663	0.81602	1.61374
H86	0.5193	0.12598	0.28423	H101	0.15232	0.84749	1.84826
H87	0.44722	0.08742	0.95889	H102	0.13429	0.79459	1.84763

Supplementary Table 2. Fractional atomic coordinates for the unit cell of COF-Ph with eclipsed stacking.

P3							
$a = b = 38.67895$, $c = 5.66787$, $\alpha = \beta = 90°$, $\gamma = 120°$; $R_{wp} = 5.90\%$, $R_p = 3.52\%$							
C1	1.60377	0.14451	-0.51724	C38	1.13508	-0.0764	-0.34346
C2	1.59637	0.17662	-0.49172	C39	1.12288	-0.04728	-0.33333
C3	1.55796	0.17118	-0.51555	C40	1.08195	-0.06036	-0.29861
C4	1.52484	0.13291	-0.56751	C41	1.05358	-0.10111	-0.28016
C5	1.5323	0.10079	-0.59692	C42	1.14996	-0.00612	-0.36424
C6	1.57094	0.10594	-0.56803	N43	1.18878	0.00971	-0.30506
C7	1.48541	0.12595	-0.58763	C44	1.22156	0.04559	-0.34246
N8	1.47528	0.15133	-0.487	N45	1.65663	0.1277	-0.54784
C9	1.43598	0.14514	-0.507	C46	1.64347	0.15293	-0.48539
C10	1.42917	0.16068	-0.29931	O47	1.17452	-0.06414	-0.37391
C11	1.3943	0.16301	-0.27251	O48	1.01544	-0.10981	-0.24896
C12	1.36616	0.15177	-0.45694	O49	1.57709	0.07418	-0.57983
C13	1.37241	0.13475	-0.66193	O50	1.55588	0.20479	-0.48693
C14	1.40688	0.13124	-0.68687	C51	1.55142	0.03277	-0.63587
C15	1.33165	0.15539	-0.42926	C52	1.55621	0.00902	-0.44597
C16	1.33383	0.19359	-0.41998	C53	1.54232	0.00919	-0.21542
C17	1.37009	0.22975	-0.4602	C54	1.54813	-0.01173	-0.0306
C18	1.29988	0.19696	-0.36149	C55	1.56831	-0.03288	-0.07361
C19	1.29511	0.11863	-0.40591	C56	1.58194	-0.03372	-0.30223
C20	1.39938	0.24625	-0.28166	C57	1.57534	-0.01342	-0.48739
C21	1.43619	0.28123	-0.31889	C58	1.18611	-0.09525	-0.42767
C22	1.44417	0.30226	-0.5338	C59	1.22824	-0.08128	-0.33023
C23	1.4144	0.28714	-0.70895	C60	1.23335	-0.09672	-0.08977
C24	1.37836	0.25088	-0.67559	C61	1.27547	-0.08365	0.00466
C25	1.28477	0.21519	-0.51498	C62	1.31246	-0.05548	-0.14253
C26	1.24858	0.21412	-0.46739	C63	1.30732	-0.04059	-0.38482
C27	1.22609	0.19461	-0.26465	C64	1.26521	-0.05367	-0.47923
C28	1.24176	0.17815	-0.10457	H65	1.61901	0.20491	-0.45513
C29	1.27848	0.17992	-0.15017	H66	1.50923	0.07299	-0.6361
C30	1.26068	0.11007	-0.53908	H67	1.46433	0.10136	-0.6766
C31	1.22423	0.07426	-0.50642	H68	1.44973	0.17042	-0.16575
C32	1.25573	0.0532	-0.21396	H69	1.38961	0.17286	-0.1157
C33	1.2919	0.08913	-0.24467	H70	1.35167	0.12472	-0.79372
N34	0.99976	-0.18895	-0.22567	H71	1.41073	0.11872	-0.83569
C35	1.03762	-0.17191	-0.30921	H72	1.39427	0.23272	-0.12169
C36	1.06519	-0.13052	-0.30069	H73	1.45678	0.2913	-0.18642
C37	1.10638	-0.11734	-0.32828	H74	1.41909	0.30281	-0.86218

H75	1.35817	0.24008	-0.81029	H90	1.52982	0.20289	-0.5047
H76	1.29906	0.22851	-0.66772	H91	1.55974	0.02643	-0.80803
H77	1.2381	0.22651	-0.58637	H92	1.52026	0.02548	-0.64419
H78	1.22605	0.16432	0.04463	H93	1.52799	0.02481	-0.18144
H79	1.28911	0.1677	-0.03065	H94	1.53785	-0.01139	0.13518
H80	1.26183	0.13004	-0.66114	H95	1.57303	-0.04767	0.06109
H81	1.19991	0.06899	-0.60444	H96	1.59676	-0.04899	-0.33385
H82	1.25431	0.03227	-0.09788	H97	1.58483	-0.01482	-0.65313
H83	1.31596	0.09346	-0.1474	H98	1.18597	-0.09954	-0.62673
H84	1.0466	-0.18996	-0.38789	H99	1.16386	-0.12474	-0.34126
H85	1.11588	-0.13762	-0.34025	H100	1.20597	-0.11777	0.01856
H86	1.07201	-0.04037	-0.28719	H101	1.27926	-0.09482	0.18355
H87	1.1401	0.01184	-0.43374	H102	1.34363	-0.04571	-0.07235
H88	1.66334	0.17963	-0.40987	H103	1.3347	-0.0199	-0.49435
H89	0.99511	-0.13803	-0.23421	H104	1.26141	-0.04284	-0.6592

Supplementary Table 3. Fractional atomic coordinates for the unit cell of COF-Na with eclipsed stacking.

P3							
$a = b = 38.65959$, $c = 5.40209$, $\alpha = \beta = 90°$, $\gamma = 120°$; $R_{wp} = 7.31\%$, $R_p = 4.69\%$							
C1	3.129	1.21043	-0.38114	O38	3.1196	1.16584	-0.73047
C2	3.11146	1.22941	-0.24716	C39	3.07496	1.2757	-0.00647
C3	3.07313	1.22334	-0.30557	C40	3.04719	1.29118	0.04034
C4	3.05052	1.19578	-0.49543	C41	3.04447	1.31757	-0.12945
C5	3.06812	1.1777	-0.63454	C42	3.01733	1.33157	-0.09212
C6	3.10663	1.18496	-0.58046	C43	2.99139	1.31802	0.11672
C7	3.43652	1.15476	-0.40721	C44	2.99442	1.29216	0.28817
C8	3.43006	1.17632	-0.59365	C45	3.02268	1.27981	0.25307
C9	3.39653	1.18153	-0.58499	C46	3.01525	1.35864	-0.25912
C10	3.36818	1.1649	-0.39221	C47	2.98721	1.37127	-0.22358
C11	3.37504	1.14342	-0.2048	C48	2.96126	1.35739	-0.01924
C12	3.40881	1.1386	-0.21115	C49	2.96331	1.33088	0.15055
C13	3.33333	1.16803	-0.39179	H50	3.12688	1.24836	-0.10545
C14	3.33377	1.20552	-0.39107	H51	3.05301	1.15857	-0.77702
C15	3.36999	1.2427	-0.38095	H52	3.44999	1.18848	-0.7363
C16	3.29809	1.2075	-0.40361	H53	3.39262	1.19721	-0.72209
C17	3.29702	1.13067	-0.39737	H54	3.35525	1.1308	-0.06264
C18	3.3796	1.27251	-0.56323	H55	3.41277	1.12273	-0.07421
C19	3.41608	1.309	-0.55525	H56	3.36012	1.2677	-0.70483
C20	3.44401	1.31668	-0.36424	H57	3.42209	1.32972	-0.69015
C21	3.43425	1.28747	-0.18017	H58	3.45395	1.29252	-0.03946
C22	3.39774	1.25122	-0.18799	H59	3.39147	1.23084	-0.05164
C23	3.2882	1.22733	-0.22033	H60	3.30728	1.24133	-0.0759
C24	3.25194	1.22783	-0.23157	H61	3.24546	1.24212	-0.09616
C25	3.22473	1.20855	-0.4272	H62	3.2158	1.17581	-0.75741
C26	3.23495	1.18973	-0.61301	H63	3.27789	1.17603	-0.74044
C27	3.27119	1.18954	-0.60219	H64	3.30898	1.11118	-0.73685
C28	3.28921	1.10359	-0.59409	H65	3.24996	1.04698	-0.74142
C29	3.25452	1.06571	-0.59668	H66	3.21383	1.07248	-0.06455
C30	3.22696	1.05338	-0.39935	H67	3.27266	1.1377	-0.0643
C31	3.23392	1.08058	-0.20651	H68	3.18081	0.9961	-0.74023
C32	3.26835	1.11877	-0.20639	H69	3.17945	1.23197	-0.14843
N33	3.19494	1.01617	-0.37947	H70	3.14572	1.16872	-0.69563
N34	3.18872	1.20604	-0.45389	H71	3.10376	1.30024	-0.06822
C35	3.17494	0.98857	-0.55681	H72	3.08021	1.2636	0.16291
C36	3.16741	1.21764	-0.31216	H73	3.06249	1.32654	-0.28235
O37	3.0582	1.24482	-0.19231	H74	2.97624	1.28232	0.44072

H75	3.025	1.26206	0.38432	C96	1.44311	1.42078	1.27787
H76	3.03396	1.36915	-0.40873	C97	1.37255	1.43878	0.7517
H77	2.98558	1.39043	-0.34744	C98	1.33112	1.42149	0.78809
H78	2.94081	1.3665	0.00581	C99	1.31296	1.3978	0.99801
H79	2.94425	1.32109	0.29883	C100	1.33618	1.39138	1.17256
C80	1.61638	1.47558	0.64518	H101	1.58468	1.48887	0.91346
C81	1.58194	1.46934	0.77741	H102	1.57079	1.39571	0.26882
C82	1.54342	1.43693	0.72479	H103	1.65668	1.52396	0.86748
C83	1.53925	1.40849	0.54318	H104	1.6419	1.42268	0.31029
C84	1.57304	1.41506	0.40563	H105	1.51814	1.48899	0.94329
C85	1.61083	1.448	0.45367	H106	1.5205	1.4595	1.18413
N86	1.67898	1.53575	0.56239	H107	1.45084	1.46442	0.72914
C87	1.65412	1.50852	0.70812	H108	1.38903	1.38665	1.47037
O88	1.50995	1.43345	0.83554	H109	1.45975	1.41735	1.41218
O89	1.64021	1.45051	0.306	H110	1.38525	1.45602	0.59797
C90	1.50492	1.45844	1.01318	H111	1.31418	1.42601	0.6608
C91	1.46159	1.44242	1.06022	H112	1.28282	1.38515	1.02368
C92	1.43772	1.44827	0.88578	H113	1.32252	1.37411	1.32486
C93	1.39624	1.43217	0.92357	N114	1.48017	1.35019	0.65786
C94	1.37783	1.40864	1.13793	C115	1.50247	1.37432	0.48833
C95	1.40165	1.40322	1.31389	H116	1.49506	1.36664	0.30632

Supplementary Table 4. Fractional atomic coordinates for the unit cell of COF-TAB-Hex with eclipsed stacking.

P1							
a = 36.9280, b = 37.7110, c = 3.8970; $\alpha = \beta$ = 90°, γ = 120°; R_p = 3.13%, R_{wp} = 2.30%							
N1	0.43118	0.95822	0.60152	N39	0.75609	0.29501	0.55015
C2	0.39496	0.93143	0.72312	C40	0.78371	0.28631	0.66551
C3	0.2227	0.55982	0.58168	C41	0.82794	0.31522	0.61611
C4	0.17891	0.53456	0.58532	C42	0.85841	0.30698	0.74823
C5	0.1589	0.4915	0.57626	C43	0.90029	0.33433	0.69859
C6	0.18463	0.47381	0.56278	C44	0.91443	0.37125	0.5254
C7	0.22861	0.49737	0.55681	C45	0.88418	0.38013	0.39482
C8	0.24668	0.54027	0.56737	C46	0.84212	0.35199	0.43914
C9	0.242	0.60374	0.59282	C47	0.4098	0.37525	0.75071
C10	0.25398	0.47835	0.54221	C48	0.38395	0.39085	0.67689
C11	0.11409	0.46675	0.58192	C49	0.33713	0.33007	0.37076
C12	0.08802	0.48099	0.43812	C50	0.36351	0.31487	0.44083
C13	0.04519	0.45719	0.44427	C51	0.40025	0.33637	0.62993
C14	0.02512	0.41792	0.59292	C52	0.45116	0.06701	0.76649
C15	0.05035	0.4036	0.73574	C53	0.43155	0.02497	0.76279
C16	0.09325	0.42674	0.73136	C54	0.45026	0.00424	0.61022
C17	0.2392	0.43913	0.38704	C55	0.48982	0.02795	0.45984
C18	0.26287	0.42029	0.38928	C56	0.51001	0.07001	0.46021
C19	0.30275	0.43888	0.54174	C57	0.64839	0.21019	0.74573
C20	0.31787	0.47762	0.69147	C58	0.6913	0.23249	0.72914
C21	0.29482	0.49709	0.69188	C59	0.71224	0.27134	0.5731
C22	0.28293	0.62899	0.73206	C60	0.68784	0.28682	0.43404
C23	0.30078	0.67087	0.74399	C61	0.64491	0.26514	0.44813
C24	0.27958	0.69057	0.62071	C62	0.50275	0.26066	0.78004
C25	0.23931	0.66621	0.48506	C63	0.48081	0.28121	0.76444
C26	0.22097	0.62433	0.46809	C64	0.42467	0.2244	0.46661
N27	0.29719	0.73328	0.6345	C65	0.44598	0.20322	0.47937
N28	0.32738	0.42003	0.55593	C66	0.48644	0.22073	0.63456
C29	0.32037	0.38627	0.40976	C67	0.62316	0.22558	0.60531
C30	0.33606	0.75962	0.57225	C68	0.49153	0.09154	0.61575
C31	0.35093	0.80308	0.61041	C69	0.55657	0.15924	0.6182
C32	0.39081	0.83226	0.4865	C70	0.57826	0.20237	0.61905
C33	0.4042	0.87358	0.52177	C71	0.55412	0.22175	0.62936
C34	0.38039	0.888	0.68436	C72	0.51007	0.19985	0.63472
C35	0.34113	0.85859	0.81304	C73	0.49026	0.15679	0.63212
C36	0.32704	0.81745	0.77049	C74	0.51261	0.13556	0.62245
O37	0.30177	0.30587	0.17647	C75	0.42764	0.31994	0.7008

O38	0.44541	0.39926	0.94437	N76	0.41823	0.2842	0.58157
C77	0.44129	0.26381	0.60689	H119	0.29391	0.36865	0.23517
C78	0.34721	0.36936	0.48735	H120	0.35875	0.75058	0.48722
C79	0.95861	0.40041	0.478	H121	0.4348	0.89612	0.41711
N80	0.98649	0.39282	0.60534	H122	0.29603	0.79566	0.87491
C81	0.26423	0.28812	0.3748	H123	0.43775	0.39206	1.17911
C82	0.24916	0.24196	0.4618	H124	0.7754	0.25778	0.80351
C83	0.20417	0.21797	0.59357	H125	0.92286	0.32642	0.80707
C84	0.18962	0.17287	0.6664	H126	0.8188	0.35875	0.33014
C85	0.14407	0.14841	0.78647	H127	0.39329	0.42157	0.77654
C86	0.13003	0.10355	0.86062	H128	0.35427	0.2843	0.33814
O87	0.8978	0.41709	0.22396	H129	0.43566	0.08171	0.89666
O88	0.84736	0.27132	0.92992	H130	0.40073	0.00785	0.88564
C89	0.86816	0.42927	0.13387	H131	0.50508	0.0127	0.33511
O90	0.31545	0.86996	0.98282	H132	0.54053	0.08706	0.33213
O91	0.41525	0.81833	0.32929	H133	0.63351	0.18036	0.87641
C92	0.45708	0.84722	0.23157	H134	0.70897	0.21915	0.8438
C93	0.48019	0.8242	0.10186	H135	0.70324	0.31691	0.30758
C94	0.52625	0.85202	0.00977	H136	0.62724	0.27821	0.32834
C95	0.54763	0.82782	-0.0967	H137	0.53327	0.2754	0.90981
C96	0.59397	0.85482	-0.18811	H138	0.49499	0.31183	0.88015
C97	0.61438	0.82975	-0.2922	H139	0.39402	0.2101	0.33902
C98	0.89166	0.47478	0.0008	H140	0.43213	0.17302	0.35564
C99	0.86339	0.49234	-0.08744	H141	0.57472	0.14345	0.61004
C100	0.88827	0.53737	-0.19678	H142	0.5703	0.25535	0.62863
C101	0.86091	0.55607	-0.28535	H143	0.45595	0.13908	0.63442
C102	0.88677	0.60105	-0.39176	H144	0.45607	0.33916	0.85132
H103	0.37407	0.93989	0.85584	H145	0.96663	0.4285	0.3343
H104	0.15944	0.54892	0.60641	H146	0.24028	0.29028	0.22213
H105	0.16971	0.4403	0.55634	H147	0.26826	0.30555	0.61257
H106	0.28087	0.55925	0.55485	H148	0.27086	0.2407	0.6497
H107	0.10224	0.51113	0.31297	H149	0.25213	0.22746	0.22616
H108	0.02689	0.46971	0.32735	H150	0.18305	0.21978	0.40279
H109	0.03557	0.37322	0.85704	H151	0.20117	0.23261	0.82855
H110	0.11157	0.41448	0.85296	H152	0.21016	0.1709	0.86212
H111	0.20845	0.42358	0.26081	H153	0.19345	0.15853	0.43286
H112	0.24972	0.39007	0.26775	H154	0.12338	0.15003	0.59016
H113	0.34871	0.49275	0.8162	H155	0.14014	0.16278	1.01925
H114	0.30755	0.52682	0.82243	H156	0.15045	0.10101	1.05324
H115	0.30033	0.61498	0.83979	H157	0.13121	0.08775	0.62705
H116	0.33207	0.68905	0.85745	H158	0.09775	0.08701	0.95888
H117	0.22213	0.68085	0.38524	H159	0.84921	0.27821	1.16778

H118	0.18981	0.60675	0.35109	H160	0.84897	0.42655	0.35947
H161	0.84699	0.40846	-0.06468	H174	0.61371	0.81025	-0.07915
H162	0.31942	0.8679	1.22287	H175	0.64728	0.84964	-0.36804
H163	0.47385	0.8667	0.45307	H176	0.9105	0.47635	-0.22458
H164	0.45647	0.8673	0.02919	H177	0.9143	0.4938	0.20029
H165	0.46289	0.80524	-0.12116	H178	0.84392	0.48954	0.137
H166	0.47743	0.80256	0.30457	H179	0.8415	0.474	-0.29348
H167	0.54299	0.87161	0.23152	H180	0.90796	0.54026	-0.41996
H168	0.52896	0.87296	-0.19908	H181	0.91005	0.55547	0.01084
H169	0.53083	0.80814	-0.31792	H182	0.84129	0.55351	-0.06262
H170	0.54463	0.80683	0.11298	H183	0.83915	0.53828	-0.49391
H171	0.61102	0.87446	0.03249	H184	0.90699	0.60463	-0.61227
H172	0.59716	0.87571	-0.399	H185	0.90714	0.61987	-0.18056
H173	0.59773	0.80954	-0.51038	H186	0.86663	0.61368	-0.46502

Supplementary Table 5. Fractional atomic coordinates for the unit cell of SIOC-COF with eclipsed stacking.

P3							
$a = b = 38.3340$, $c = 4.7587$, $\alpha = \beta = 90°$, $\gamma = 120°$; $R_{wp} = 3.94\%$, $R_p = 2.61\%$							
N1	0.80984	-1.97772	0.30836	C38	1.6273	-1.23473	0.75531
C2	0.82945	-1.95117	0.50276	N39	1.52254	-1.31823	0.62146
C3	1.1713	-1.66594	0.57251	C40	1.51138	-1.34417	0.82554
C4	1.20678	-1.66667	0.57253	C41	1.47564	-1.38348	0.78912
C5	1.12998	-1.70324	0.547	C42	1.44713	-1.39061	0.57713
C6	1.2468	-1.63073	0.64707	C43	1.4135	-1.42804	0.53763
C7	1.12121	-1.7399	0.6736	C44	1.40584	-1.46096	0.70948
C8	1.08214	-1.77194	0.68169	C45	1.43407	-1.45383	0.92351
C9	1.05066	-1.7695	0.55233	C46	1.46779	-1.41635	0.96278
C10	1.06005	-1.73454	0.4005	H47	0.81589	-1.95294	0.71008
C11	1.09887	-1.70186	0.39926	H48	1.14427	-1.74372	0.78153
C12	1.24953	-1.60676	0.87769	H49	1.0758	-1.79933	0.79628
C13	1.28564	-1.57276	0.94471	H50	1.03612	-1.73205	0.29208
C14	1.32066	-1.56235	0.79129	H51	1.10425	-1.67435	0.29067
C15	1.31835	-1.58769	0.5701	H52	1.2225	-1.61439	1.00079
C16	1.28217	-1.62157	0.49973	H53	1.2868	-1.55369	1.12049
N17	1.35655	-1.52709	0.85873	H54	1.34533	-1.58052	0.4466
N18	1.01111	-1.80141	0.57724	H55	1.28166	-1.64005	0.32138
C19	0.99302	-1.82272	0.35589	H56	1.00948	-1.81783	0.15648
C20	0.95122	-1.85445	0.38079	H57	0.93733	-1.82748	0.7359
C21	0.92583	-1.85319	0.58991	H58	0.86904	-1.88131	0.79707
C22	0.88667	-1.8841	0.62484	H59	0.88346	-1.94495	0.09153
C23	0.87014	-1.91842	0.45173	H60	0.95194	-1.89063	0.02817
C24	0.8949	-1.91893	0.23456	H61	1.35369	-1.50523	0.4617
C25	0.93407	-1.88786	0.19878	H62	1.75277	-0.98895	0.70944
C26	1.37001	-1.49982	0.6625	H63	1.69345	-1.05196	0.82331
C27	1.77372	-1.01313	0.37418	H64	1.72154	-1.11106	0.15673
C28	1.74694	-1.01545	0.58546	H65	1.7831	-1.04704	0.04929
C29	1.71266	-1.05155	0.65024	H66	1.58857	-1.18709	0.36562
C30	1.70361	-1.08683	0.50275	H67	1.52997	-1.25366	0.3771
C31	1.72872	-1.08415	0.27985	H68	1.5946	-1.29628	0.88464
C32	1.76328	-1.04812	0.2197	H69	1.65464	-1.23052	0.86028
C33	1.6275	-1.20314	0.60306	H70	1.52897	-1.33685	1.02154
C34	1.59116	-1.21049	0.47905	H71	1.45139	-1.36604	0.43561
C35	1.5574	-1.24845	0.48617	H72	1.39274	-1.43121	0.36674
C36	1.55749	-1.28027	0.62918	H73	1.4296	-1.47853	1.06444
C37	1.59297	-1.27239	0.76906	H74	1.48857	-1.41308	1.1337

Section I. ^{1}H NMR and ^{13}C NMR spectra.

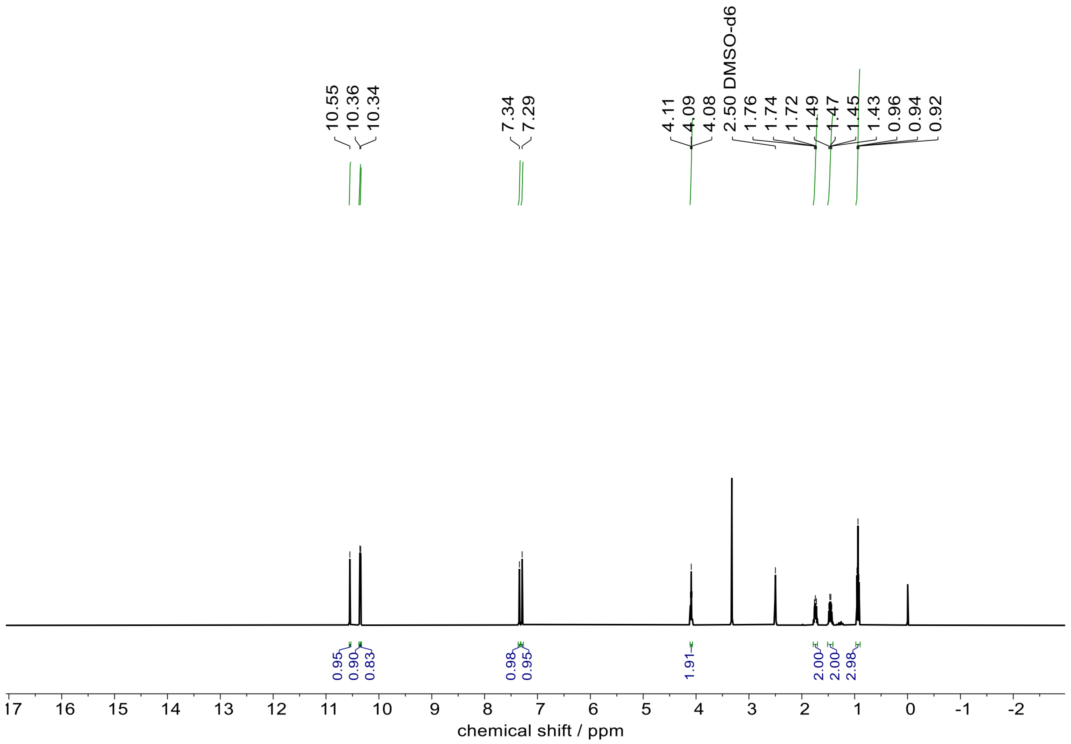

Supplementary Fig. 43 ^{1}H NMR (400 MHz, DMSO-d_6) of TPA-Bu.

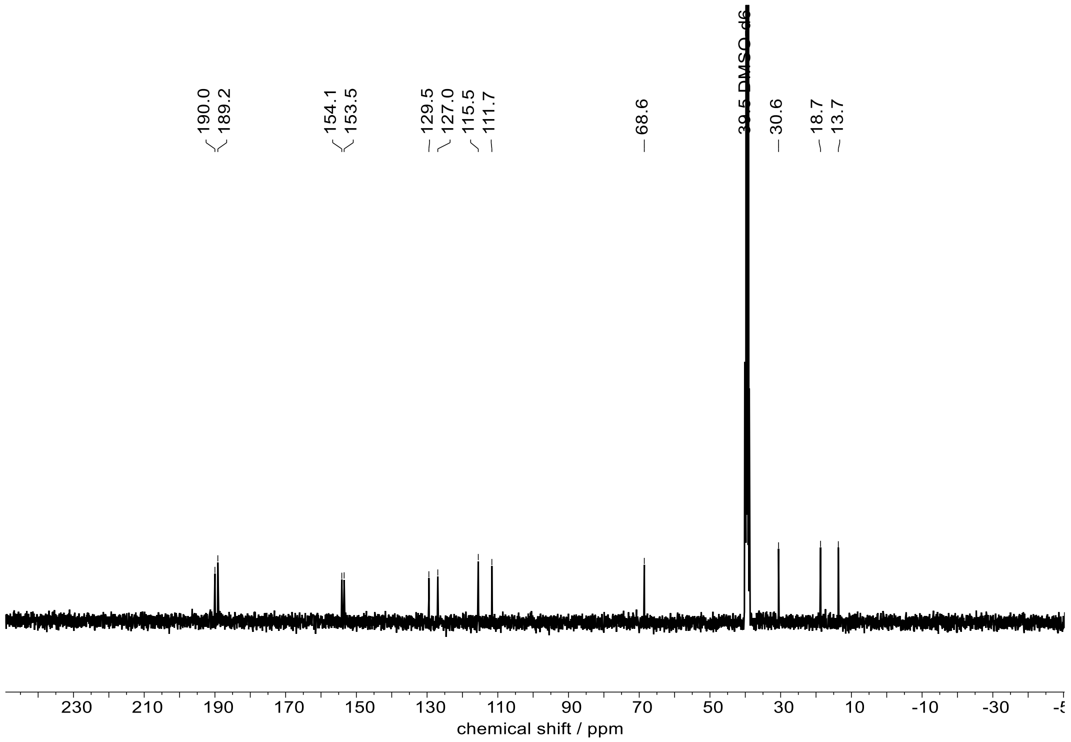

Supplementary Fig. 44 ^{13}C NMR (101 MHz, DMSO-d_6) of TPA-Bu.

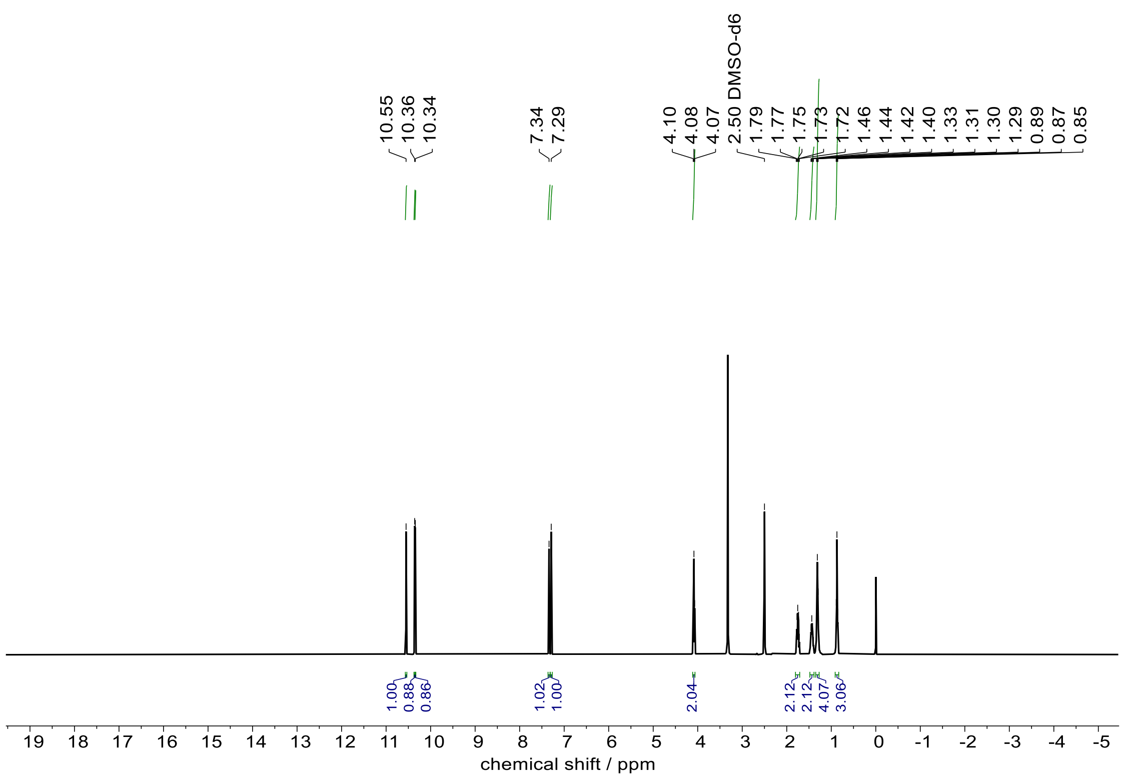

Supplementary Fig. 45 ^{1}H NMR (400 MHz, DMSO-d_6) of TPA-Hex.

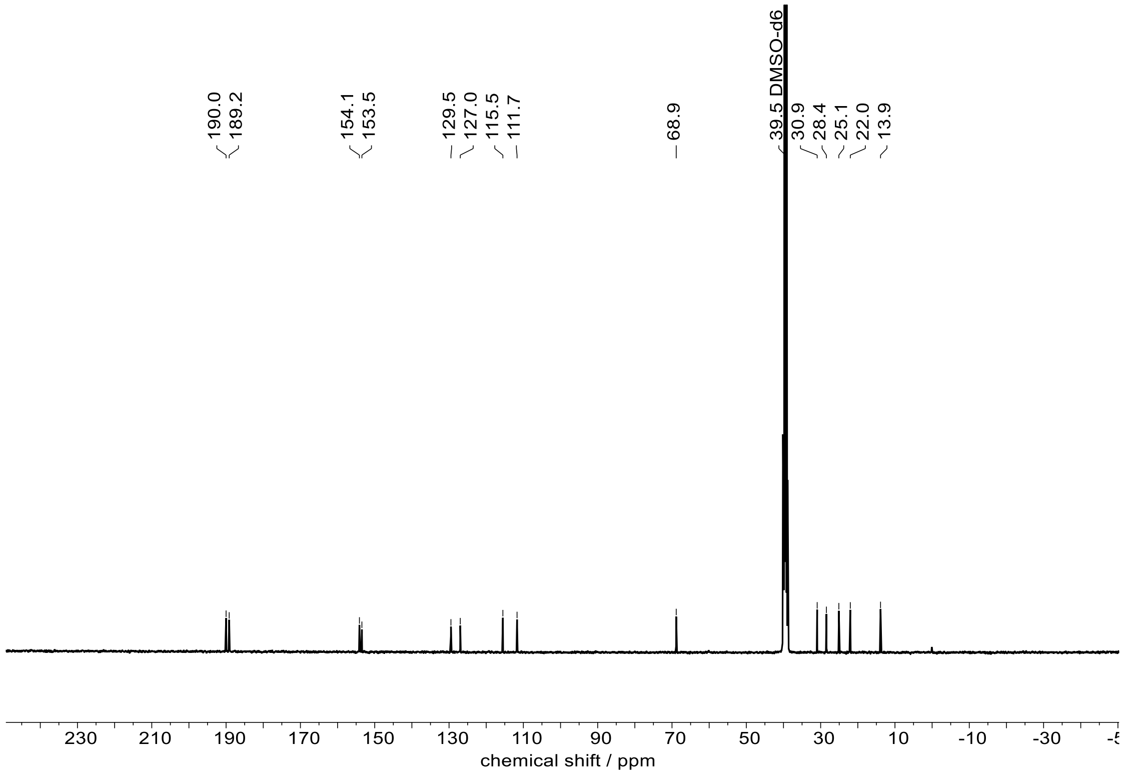

Supplementary Fig. 46 ^{13}C NMR (101 MHz, DMSO-d_6) of TPA-Hex.

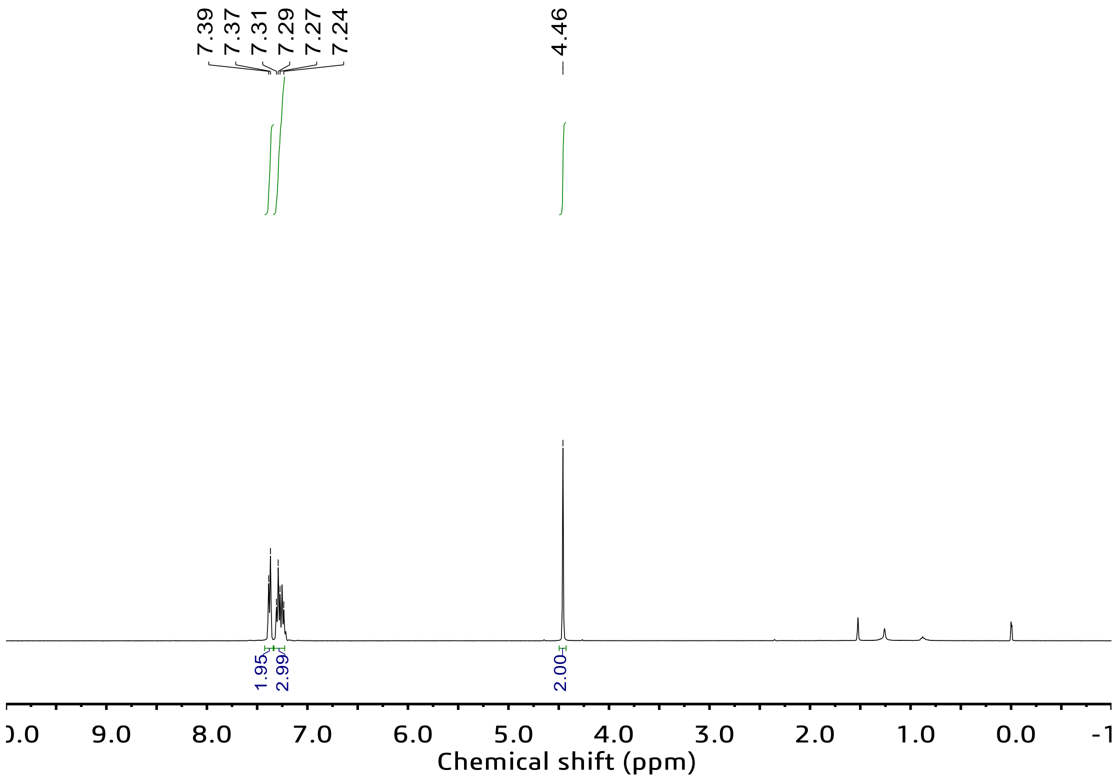

Supplementary Fig. 47 ^{1}H NMR (400 MHz, $CDCl_3$-*d*) of Benzyl iodide.

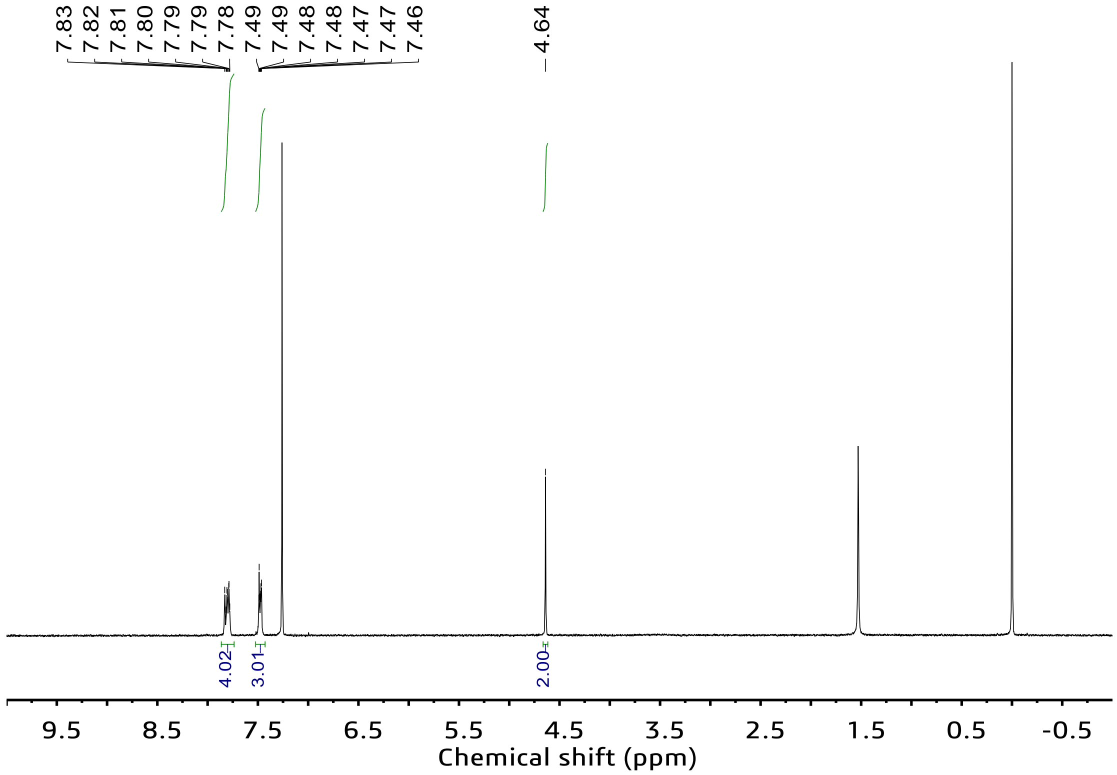

Supplementary Fig. 48 ^{1}H NMR (400 MHz, $CDCl_3$-*d*) of 2-(Iodomethyl)naphthalene.

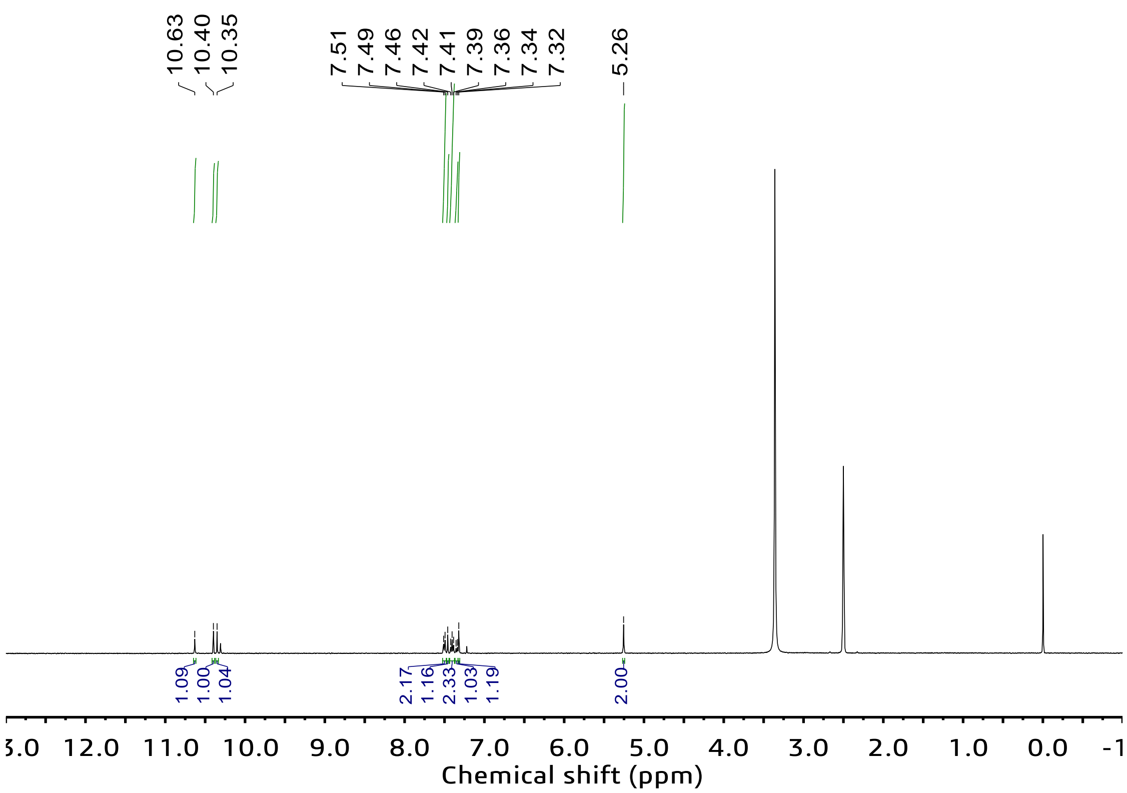

Supplementary Fig. 49 ^{1}H NMR (400 MHz, DMSO-d_6) of TPA-Ph.

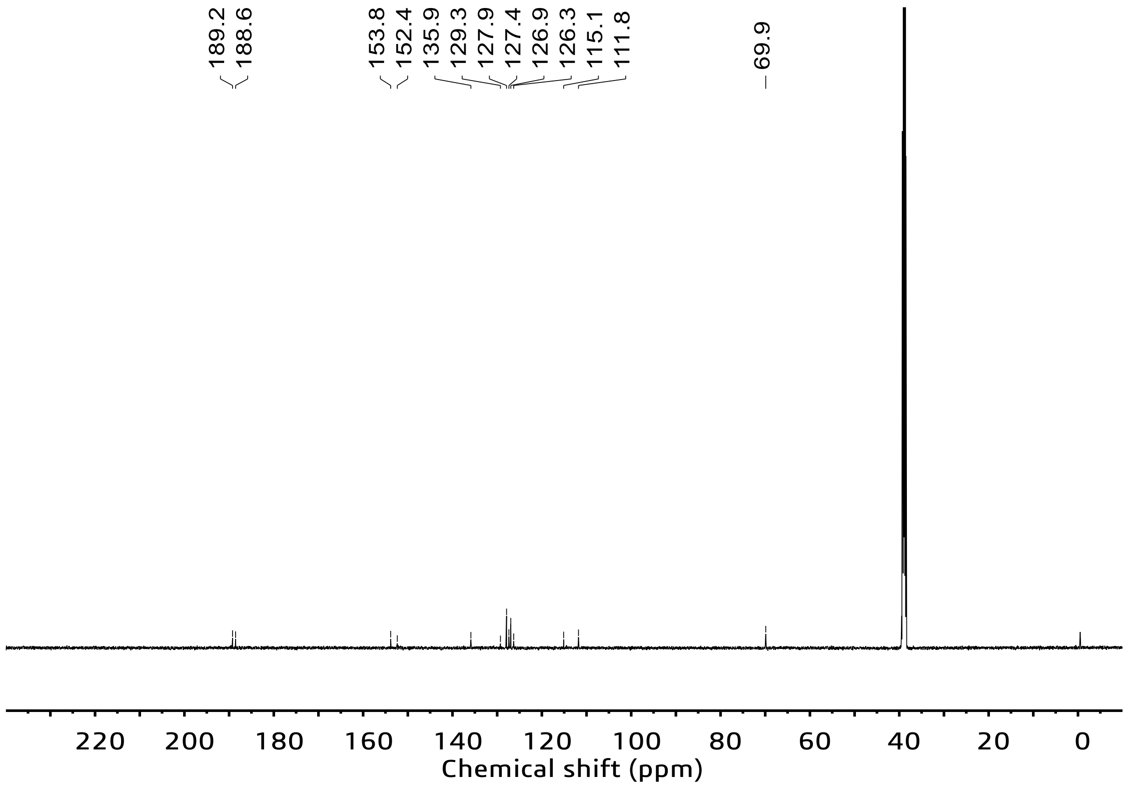

Supplementary Fig. 50 ^{13}C NMR (101 MHz, DMSO-d_6) of TPA-Ph.

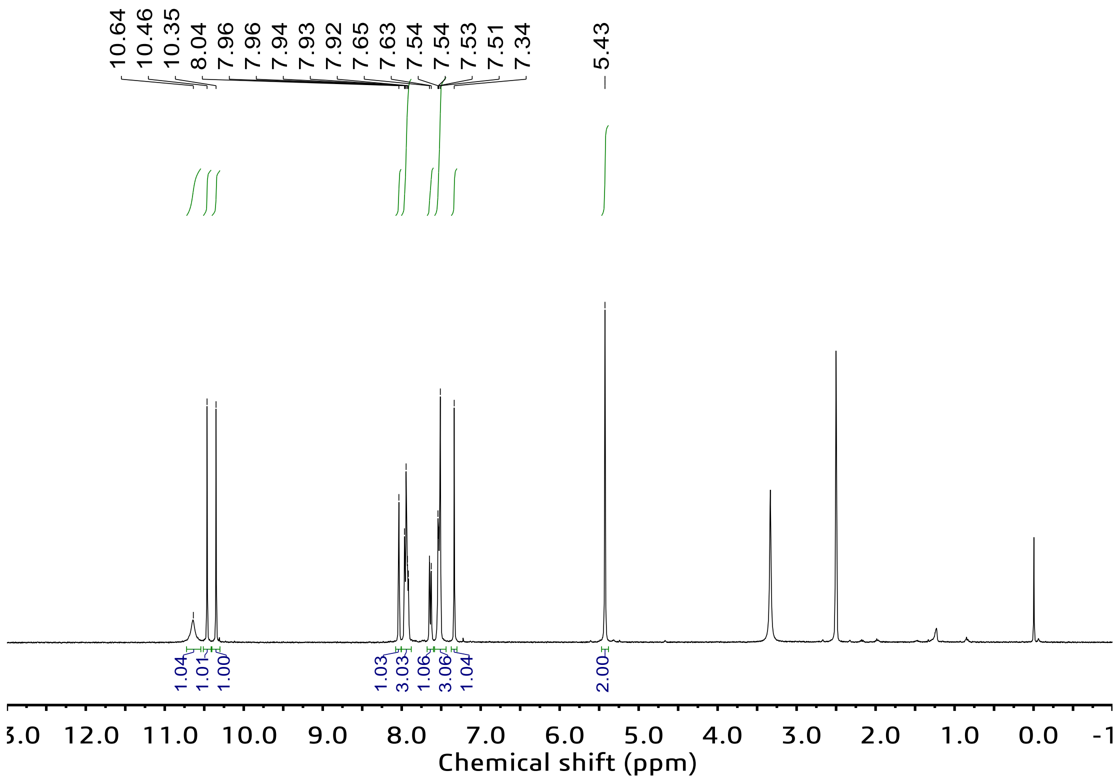

Supplementary Fig. 51 ^{1}H NMR (400 MHz, DMSO-d_6) of TPA-Na.

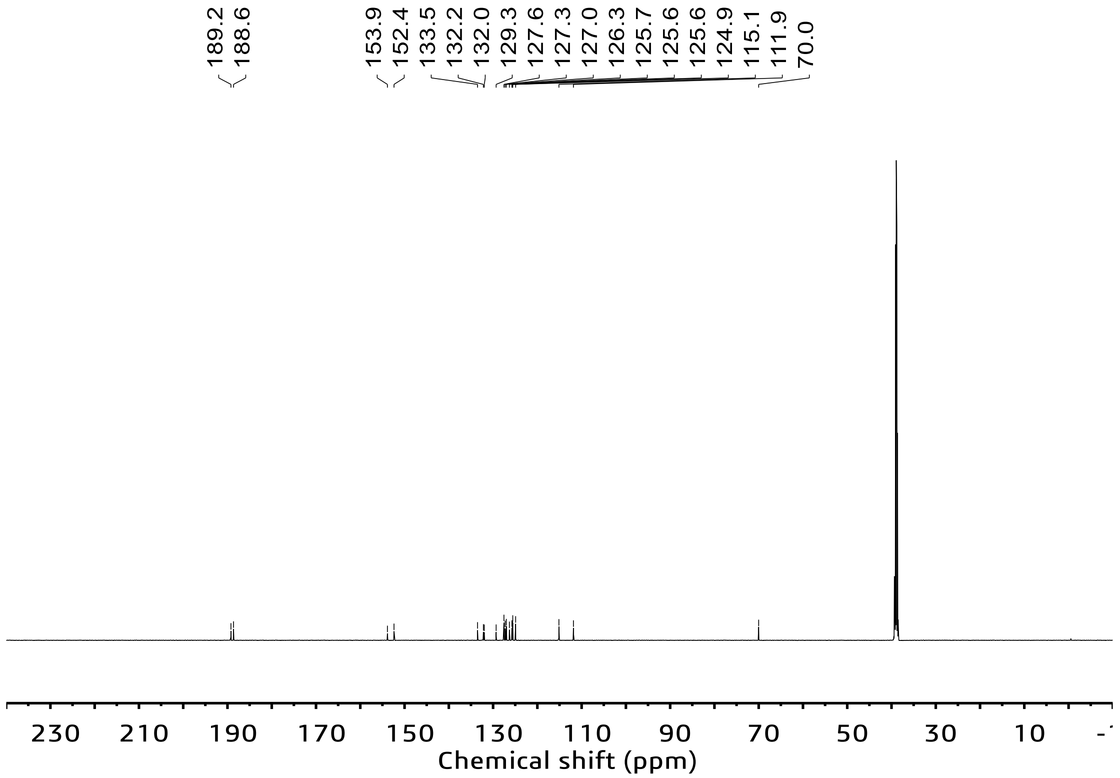

Supplementary Fig. 52 ^{13}C NMR (101 MHz, DMSO-d_6) of TPA-Na.

Supplementary References.

1. Fan, Z. et al. $NaBH_4/I_2$-Mediated Efficient Iodination of Alcohols. *Chin. J. Org. Chem.*, **39**, 2333-2337 (2019).
2. Zhou, T.-Y. et al. One-Step Construction of Two Different Kinds of Pores in A 2D Covalent Organic Framework. *J. Am. Chem. Soc.* **136**, 15885-15888 (2014).
3. Niklas K. et al. Enforcing Extended Porphyrin J-Aggregate Stacking in Covalent Organic Frameworks. *J. Am. Chem. Soc*, **140**, 16544-16552 (2018).
4. Niaz A. K. et al. Assembling covalent organic framework membranes via phase switching for ultrafast molecular transport. *Nat. Comm.***13**, 3169 (2022).
